# Supplementary material for: Homobivalent Lamellarin-Like Schiff Bases: In Vitro Evaluation of Their Cancer Cell Cytotoxicity and Multitargeting Anti-Alzheimer’s Disease Potential
Source: Molecules. 2021 Jan 12;26(2):359. doi: 10.3390/molecules26020359 (PMC7827648; doi:10.3390/molecules26020359)
Supplement: Supplementary file 1 [file molecules-26-00359-s001.pdf]

Homobivalent lamellarin-like Schiff bases as prospective multitarget anti-Alzheimer's disease agents

Alisa A. Nevskaya,[1] Lada V. Anikina,[2] Rosa Purgatorio,[3] Marco Catto,[3] Orazio Nicolotti,[3] Modesto de Candia,[3] Leonardo Pisani,[3] Tatiana N. Borisova,[1] Almira R. Miftyahova,[1] Aleksey V. Varlamov,[1] Elena Yu. Nevskaya, [1] Roman S. Borisov,[1,4,5] Leonid G. Voskressensky[1] and Cosimo D. Altomare[3] *

[1] Organic Chemistry Department, Peoples' Friendship University of Russia (RUDN University), 6 Miklukho-Maklaya St., Moscow, 117198, Russian Federation

[2] Institute of Physiologically Active Compounds, Russian Academy of Sciences, Chernogolovka, Russian Federation

[3] Department of Pharmacy-Pharmaceutical Sciences, University of Bari Aldo Moro, Via E. Orabona 4, 70125 Bari, Italy

[4] Topchiev Institute of Petrochemical Synthesis, Russian Academy of Sciences, 29 Leninskii prosp., Moscow, 119991, Russian Federation

[5] Lomonosov North (Arctic) Federal University, Severnaya Dvina Emb. 17, Arkhangelsk, 163002, Russian Federation

* Correspondence: cosimodamiano.altomare@uniba.it; Tel.: +39-080-5442781

SUPPORTING INFORMATION

Copies of ^{1}H and ^{13}C NMR spectra S2

Multi-fingerprint Similarity Search aLgorithm (MuSSeL) outputs S36

Copies of ^{1}H and ^{13}C NMR spectra

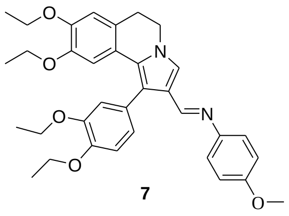

(*E*)-1-(1-(3,4-diethoxyphenyl)-8,9-diethoxy-5,6-dihydropyrrolo[2,1-*a*]isoquinolin-2-yl)-N-(4-methoxyphenyl)methanimine

^{1}H NMR (600 MHz, $CDCl_3$)

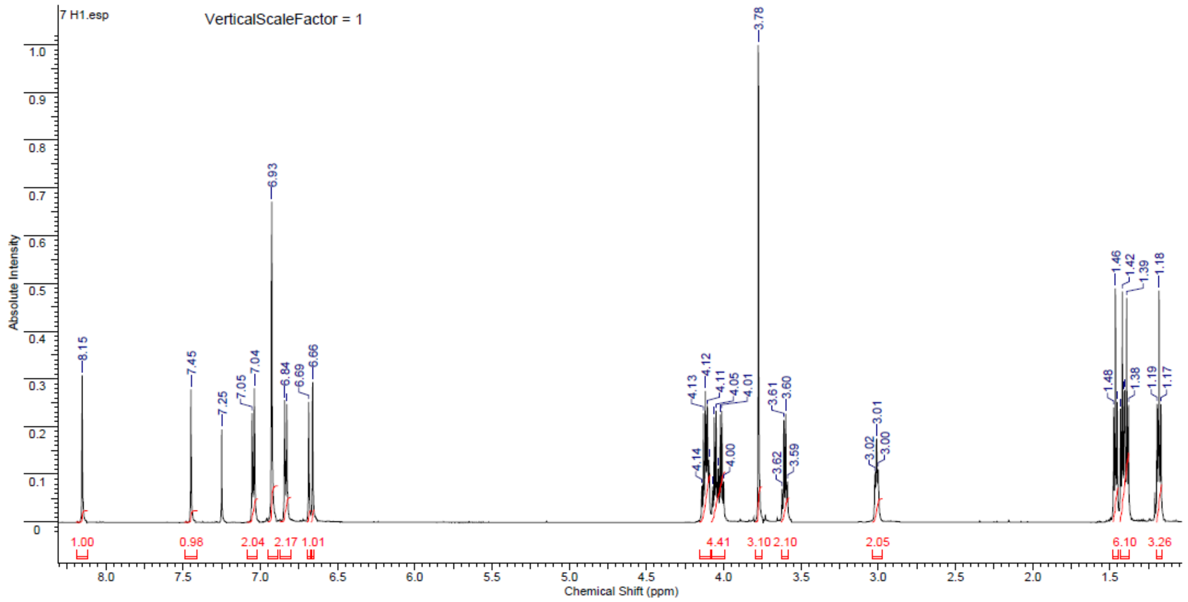

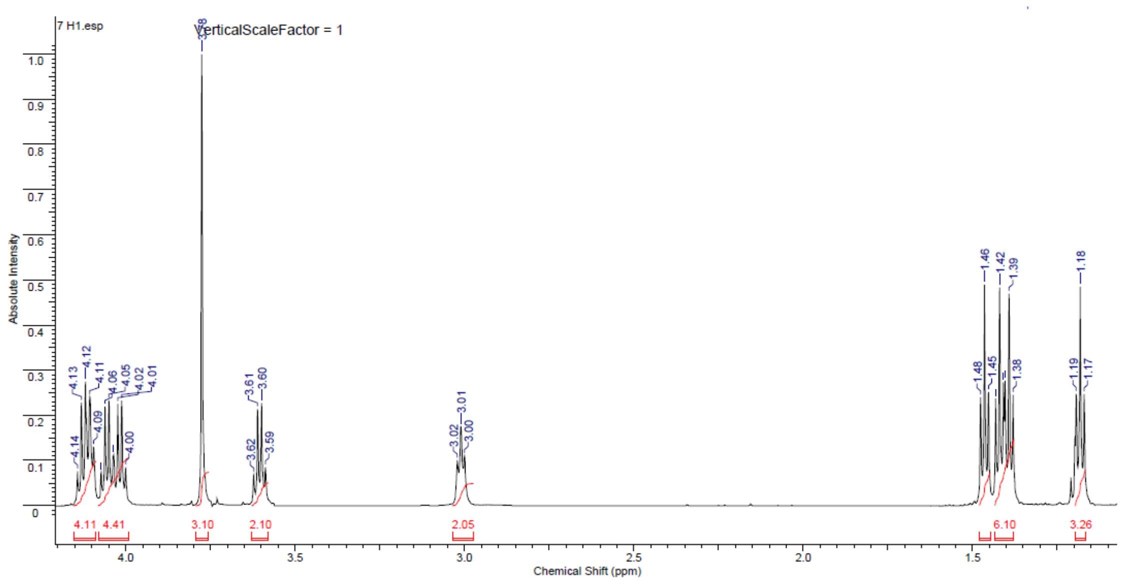

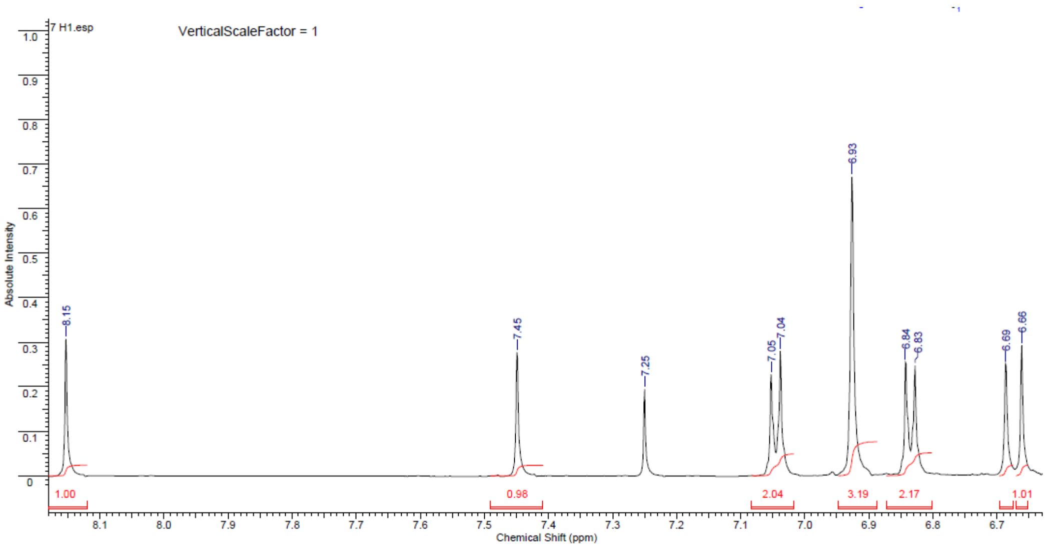

^{13}C NMR (150 MHz, $CDCl_3$)

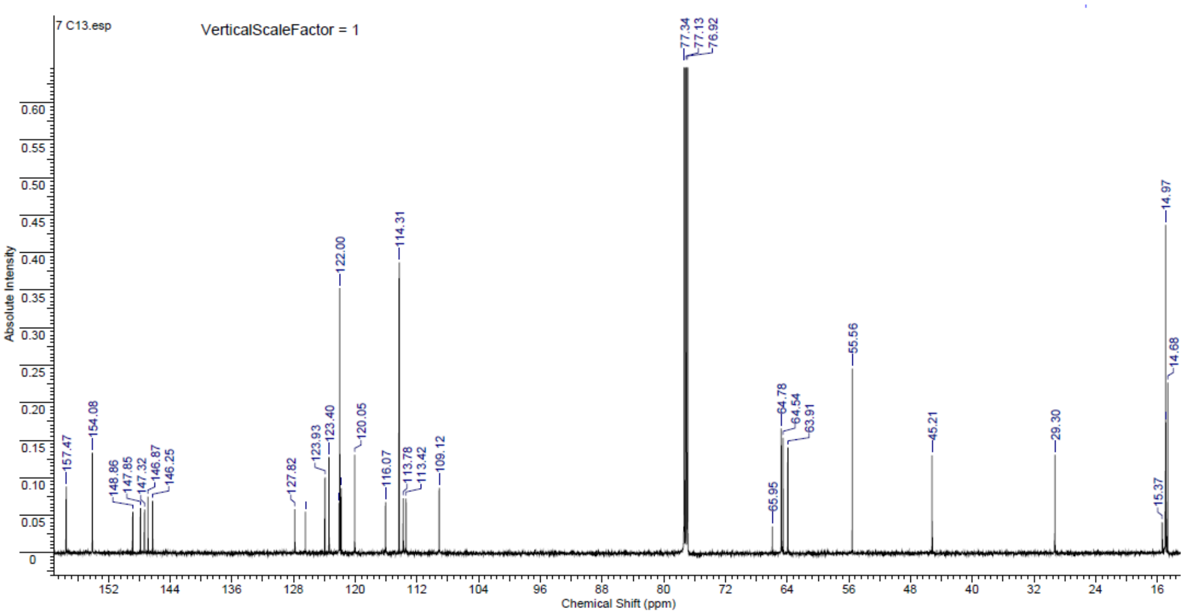

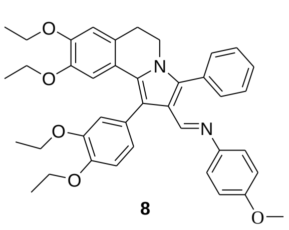

(*E*)-1-(1-(3,4-diethoxyphenyl)-8,9-diethoxy-3-phenyl-5,6-dihydropyrrolo[2,1-*a*]isoquinolin-2-yl)-N-(4-methoxyphenyl)methanimine

^{1}H NMR (600 MHz, $CDCl_3$)

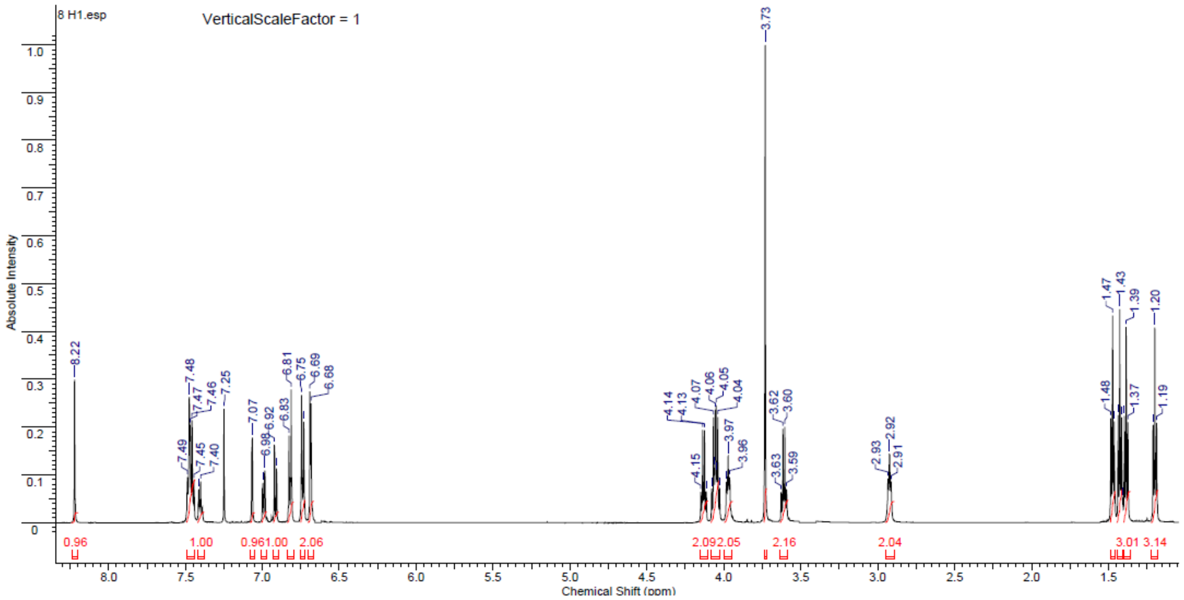

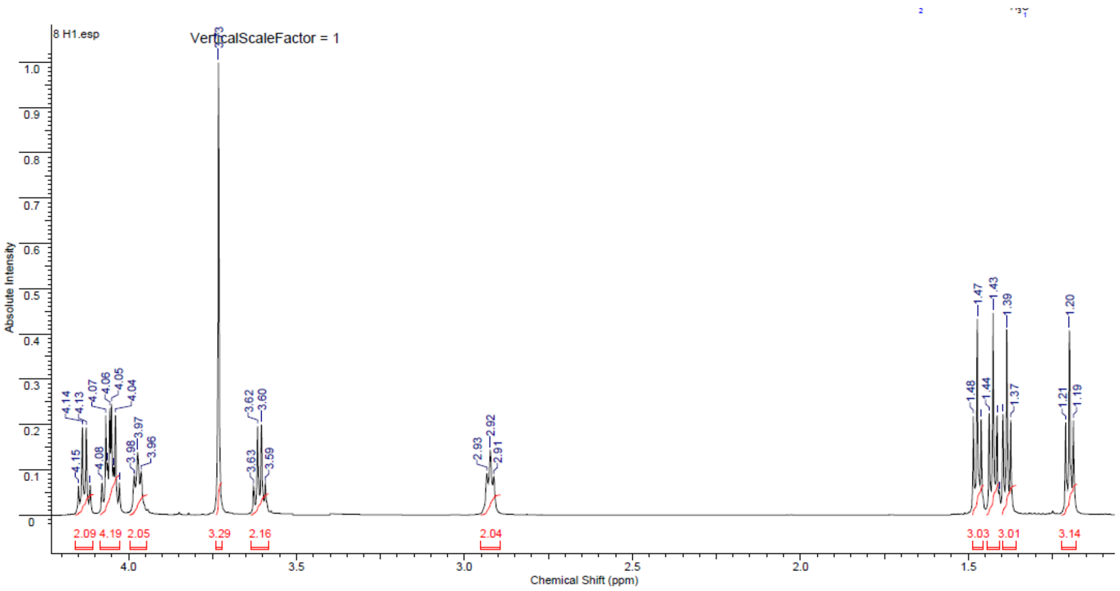

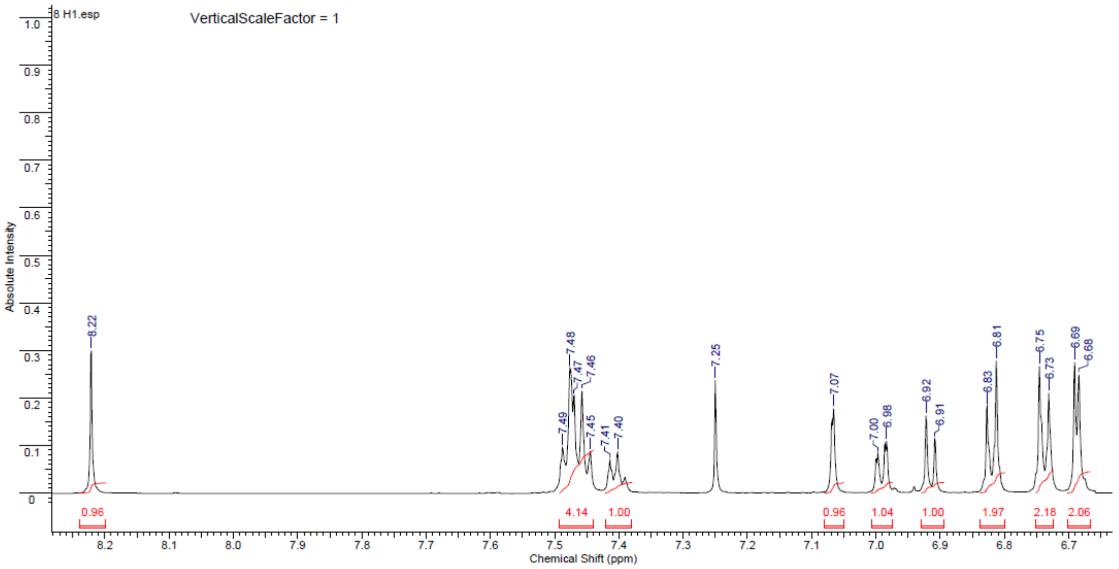

^{13}C NMR (150 MHz, $CDCl_3$)

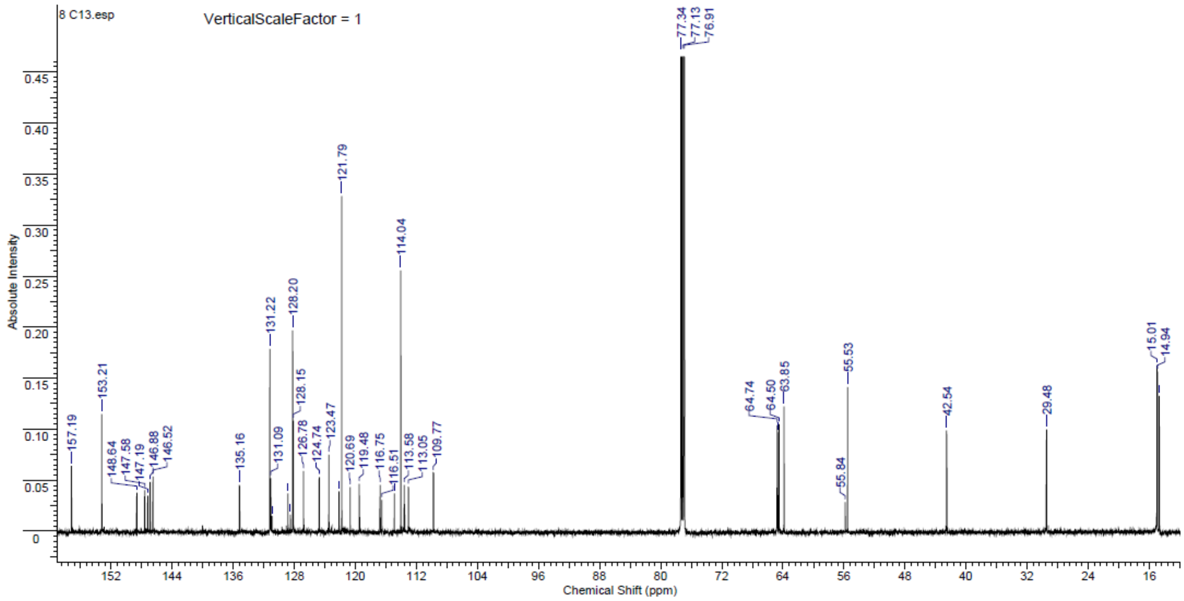

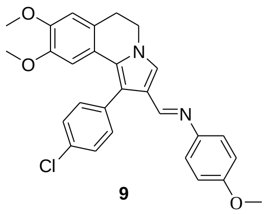

9

(*E*)-1-(1-(4-chlorophenyl)-8,9-dimethoxy-5,6-dihydropyrrolo[2,1-*a*]isoquinolin-2-yl)-N-(4-methoxyphenyl)methanimine

^{1}H NMR (600 MHz, $CDCl_3$)

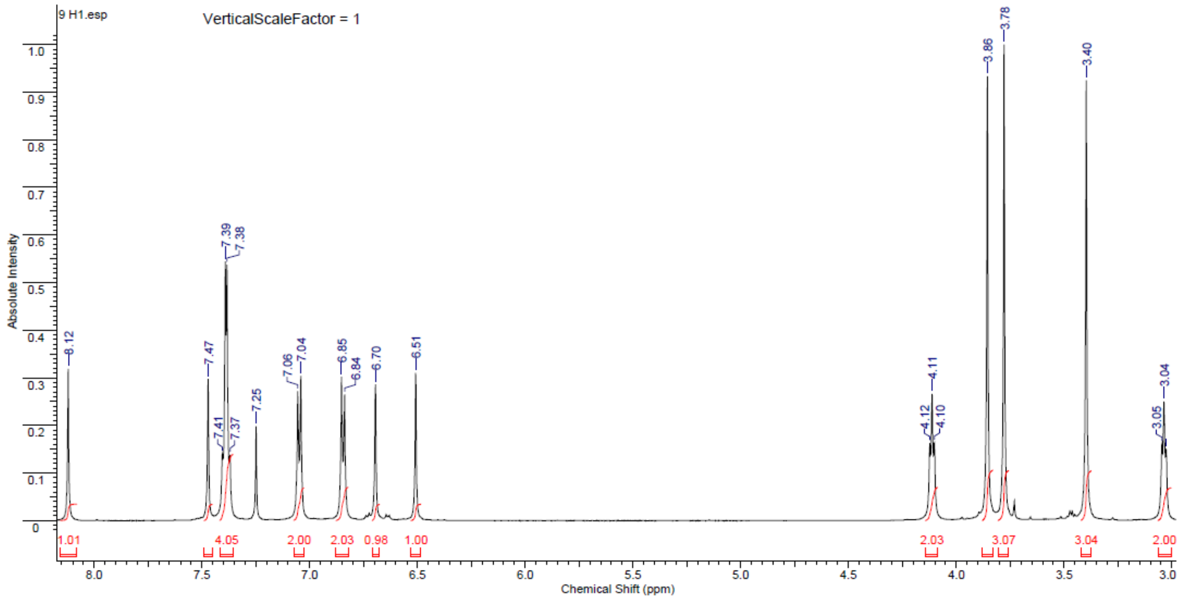

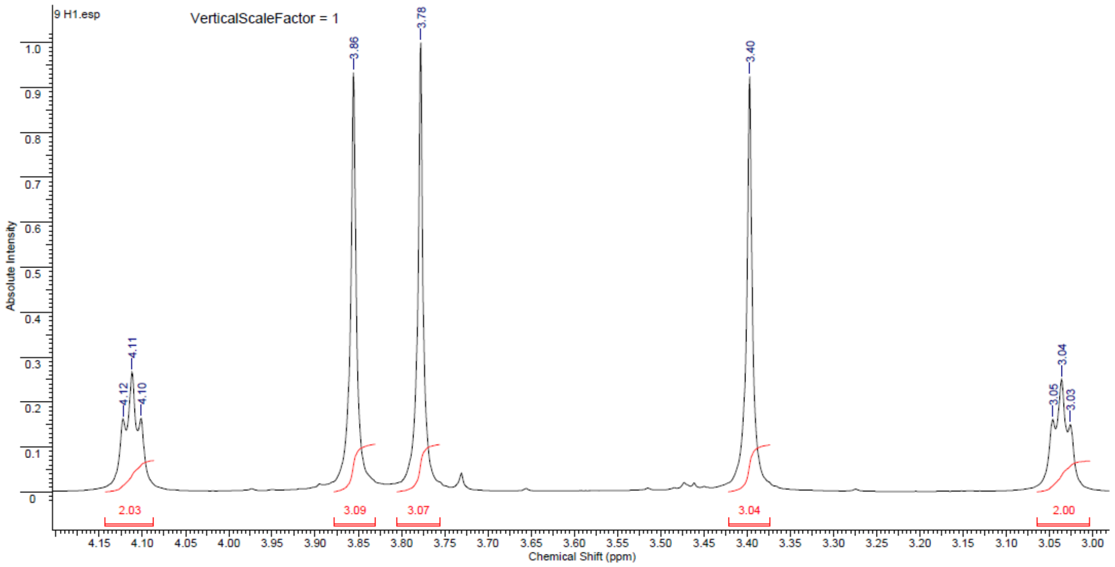

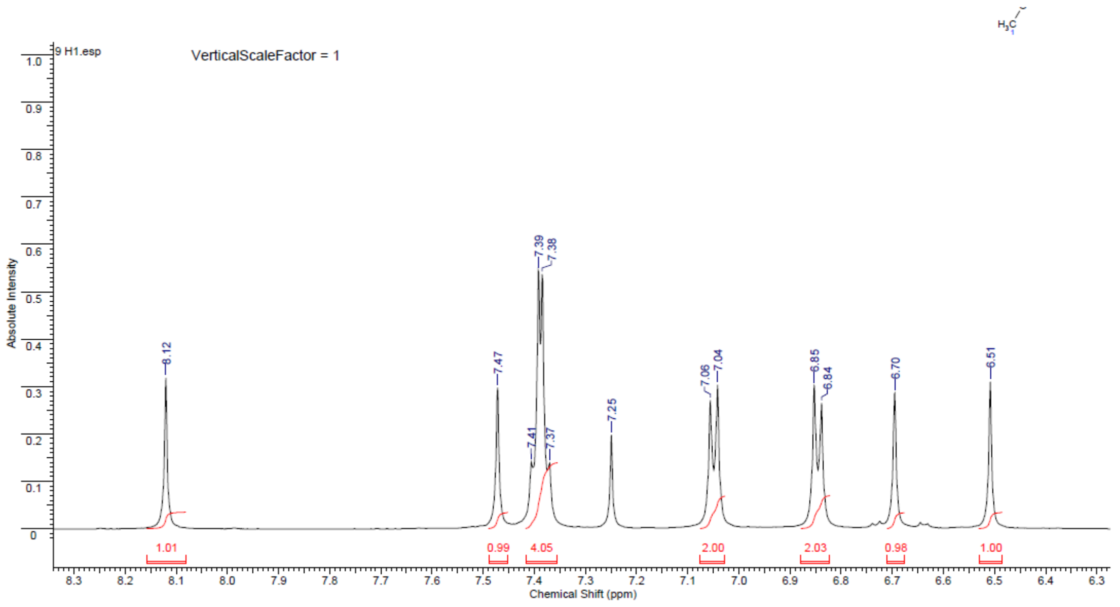

^{13}C NMR (150 MHz, $CDCl_3$)

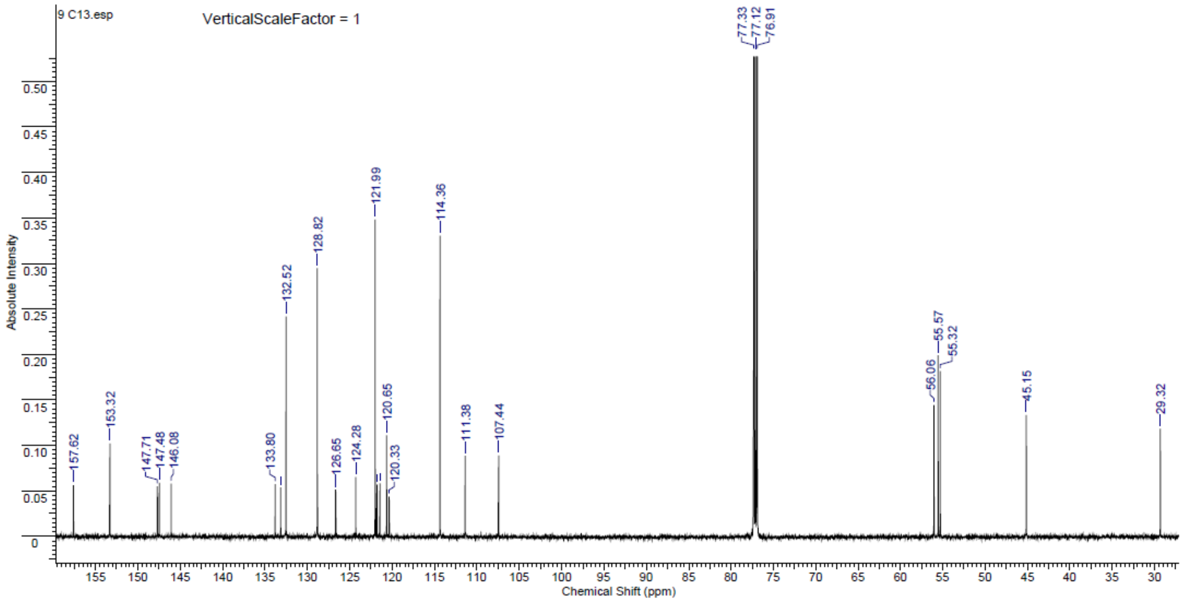

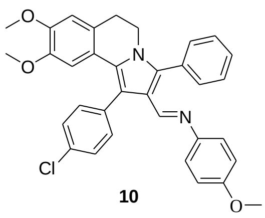

10

(*E*)-1-(1-(4-chlorophenyl)-8,9-dimethoxy-3-phenyl-5,6-dihydropyrrolo[2,1-*a*]isoquinolin-2-yl)-N-(4-methoxyphenyl)methanimine

^{1}H NMR (600 MHz, $CDCl_3$)

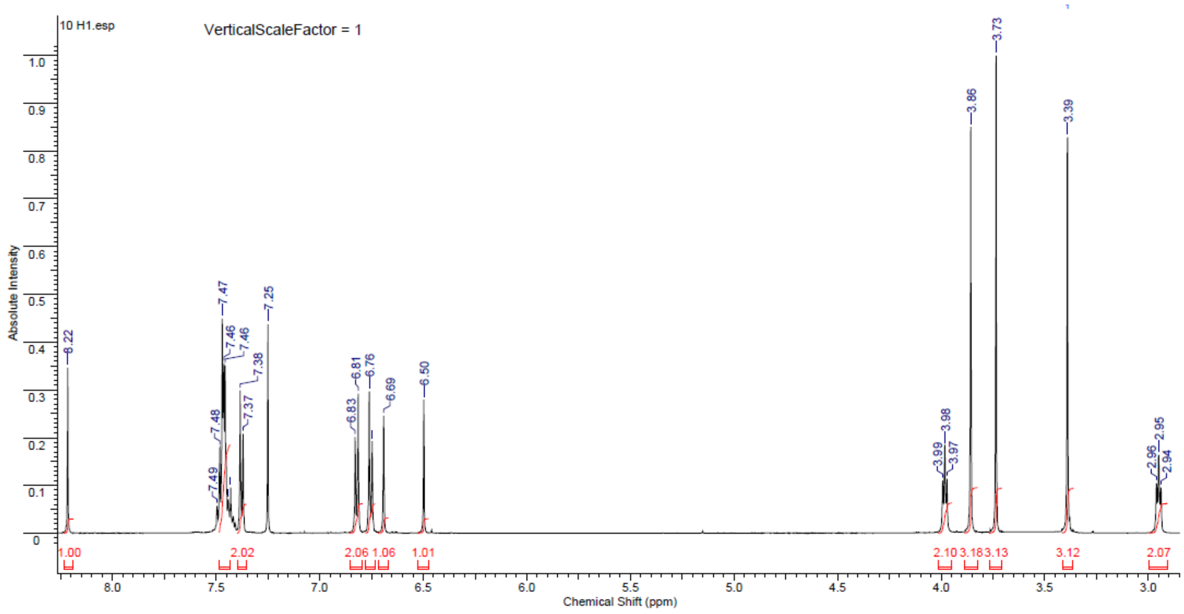

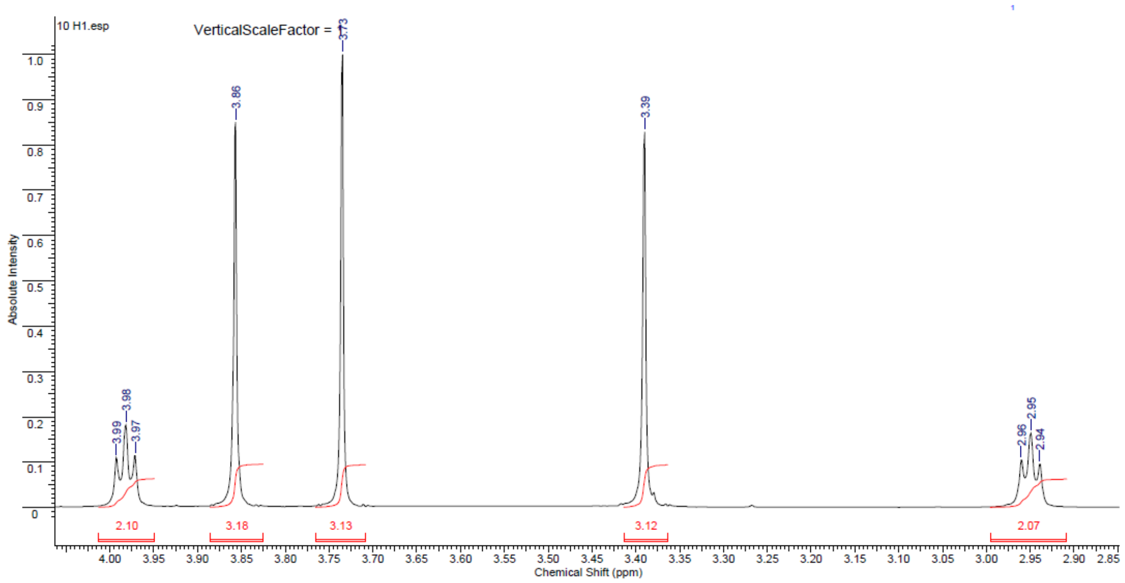

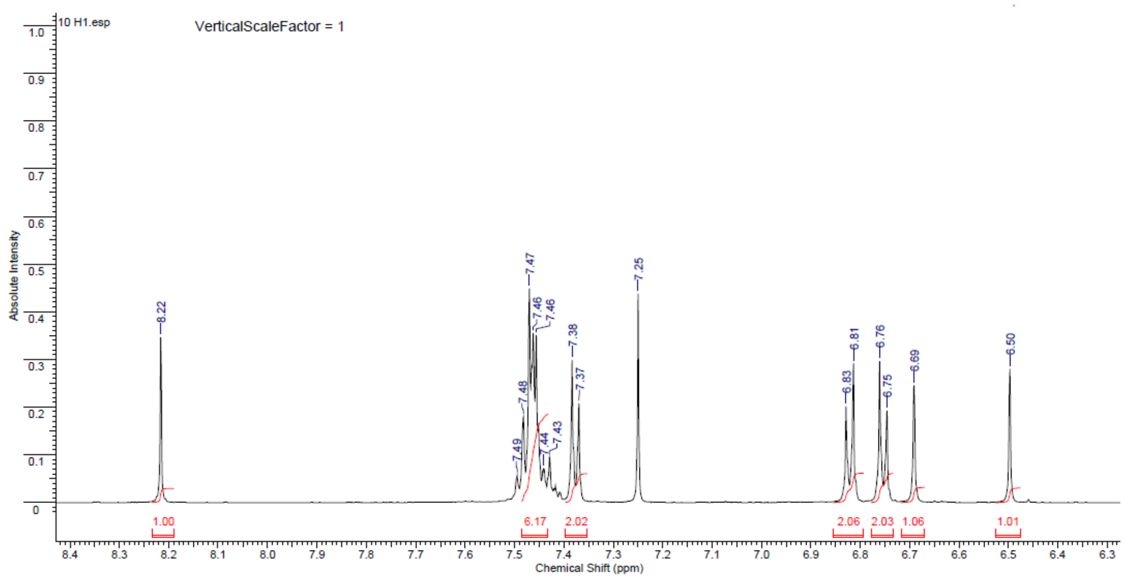

^{13}C NMR (150 MHz, $CDCl_3$)

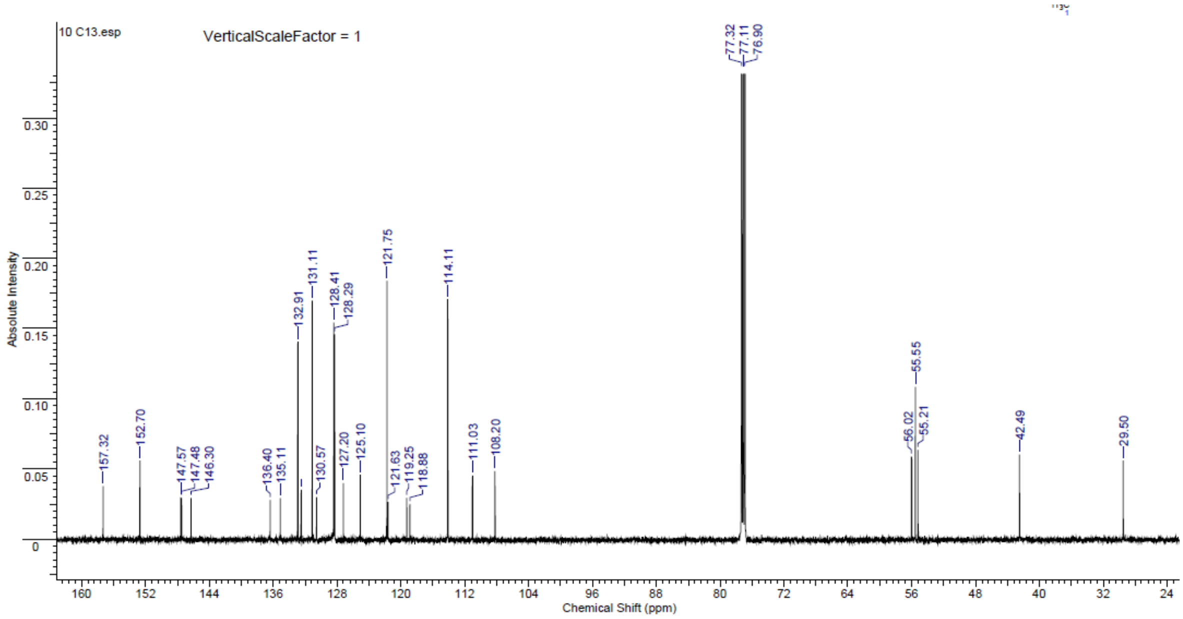

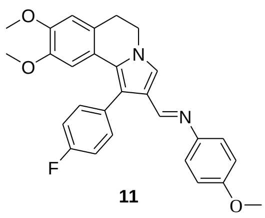

11

(*E*)-1-(1-(4-fluorophenyl)-8,9-dimethoxy-5,6-dihydropyrrolo[2,1-*a*]isoquinolin-2-yl)-N-(4-methoxyphenyl)methanimine

^{1}H NMR (600 MHz, $CDCl_3$)

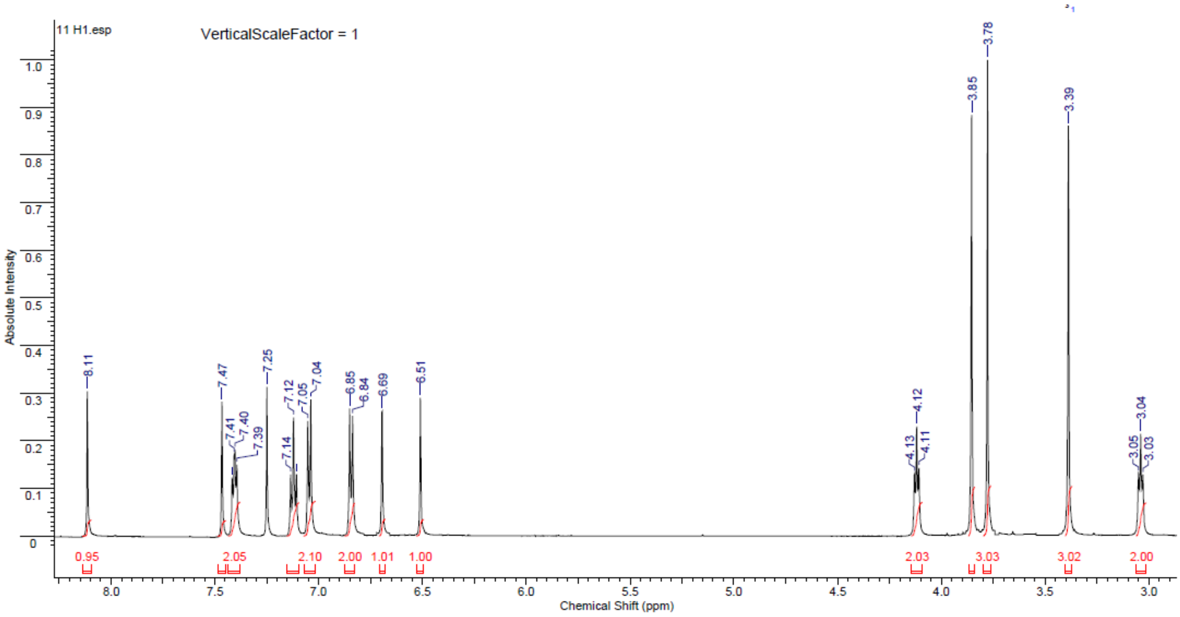

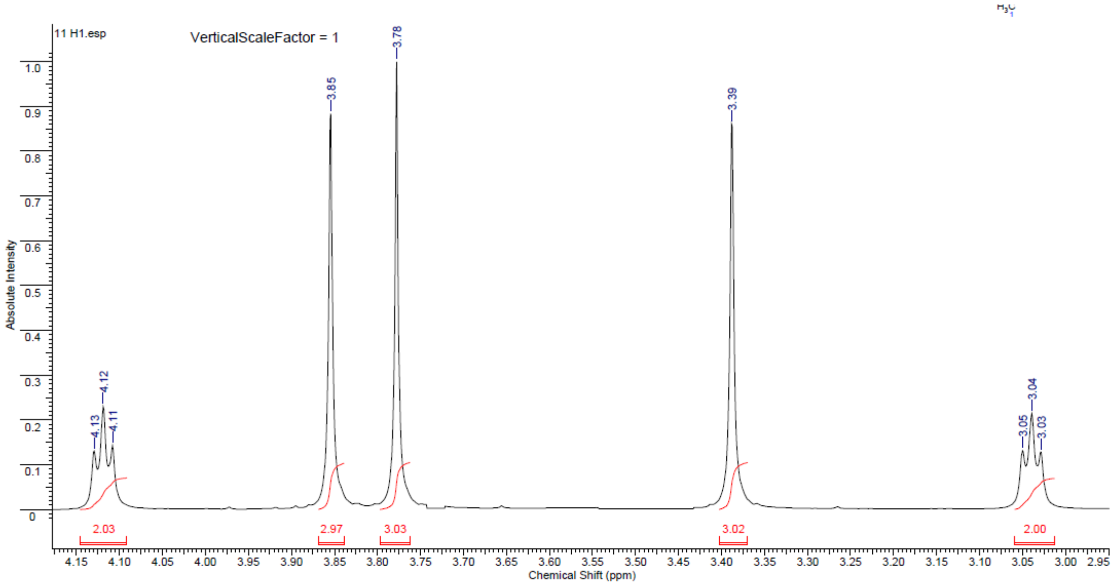

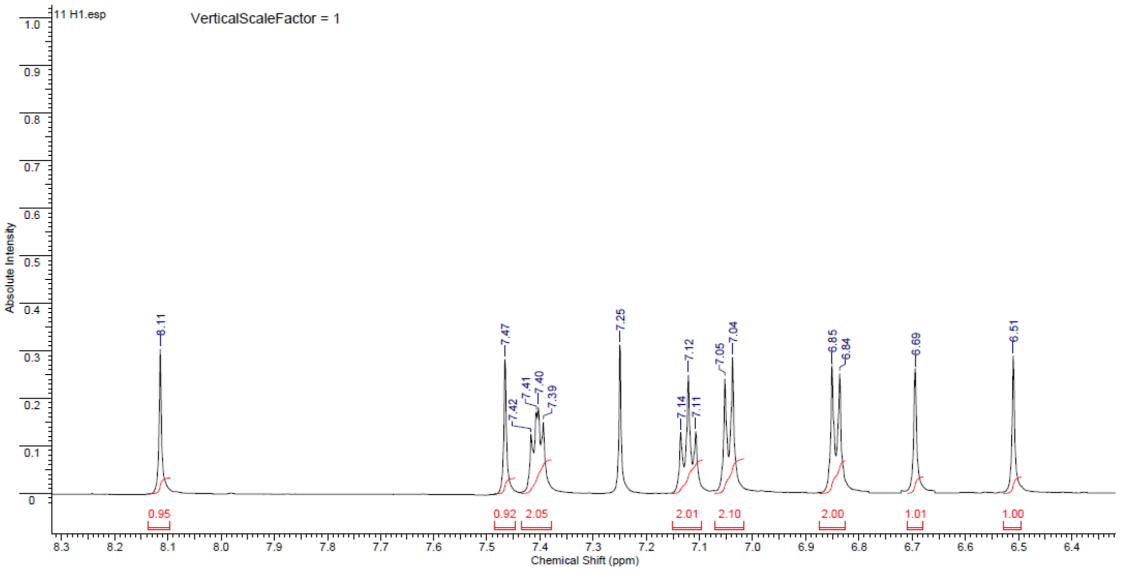

^{13}C NMR (150 MHz, $CDCl_3$)

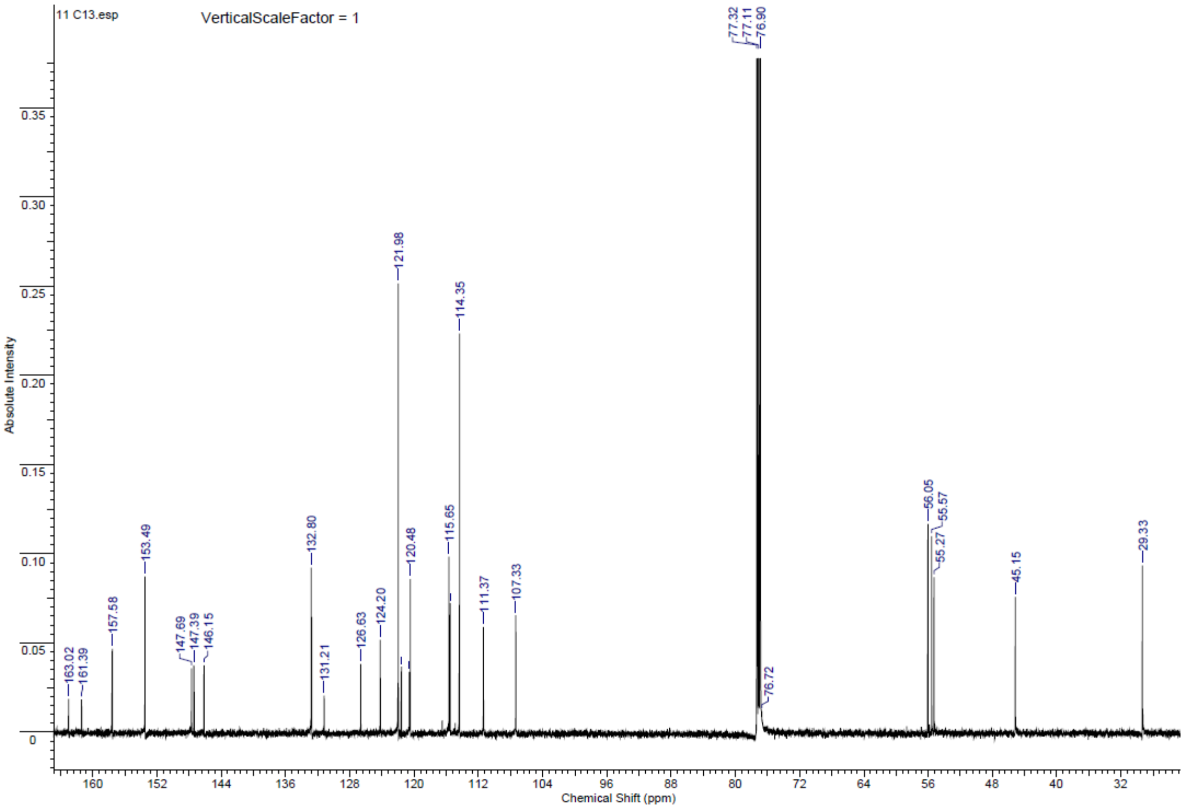

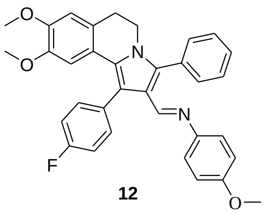

12

(*E*)-1-(1-(4-fluorophenyl)-8,9-dimethoxy-3-phenyl-5,6-dihydropyrrolo[2,1-*a*]isoquinolin-2-yl)-N-(4-methoxyphenyl)methanimine

^{1}H NMR (600 MHz, $CDCl_3$)

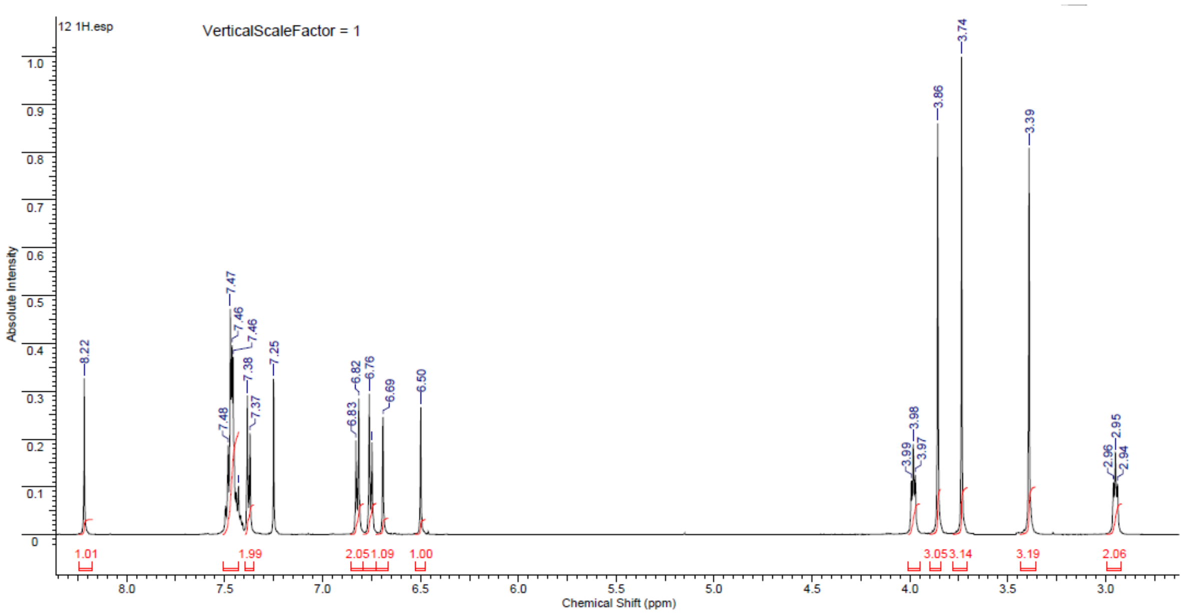

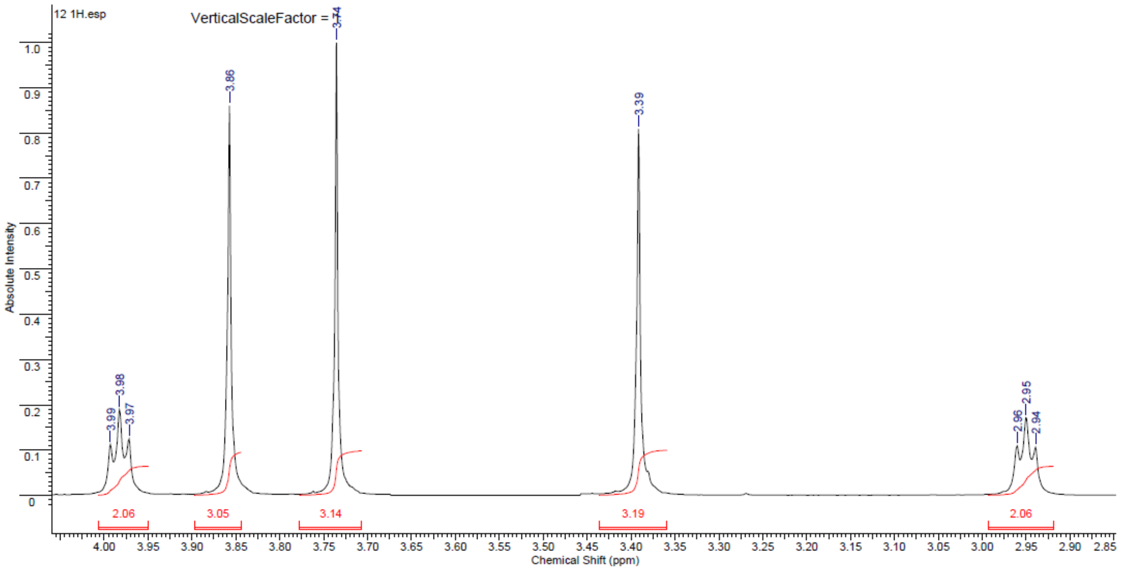

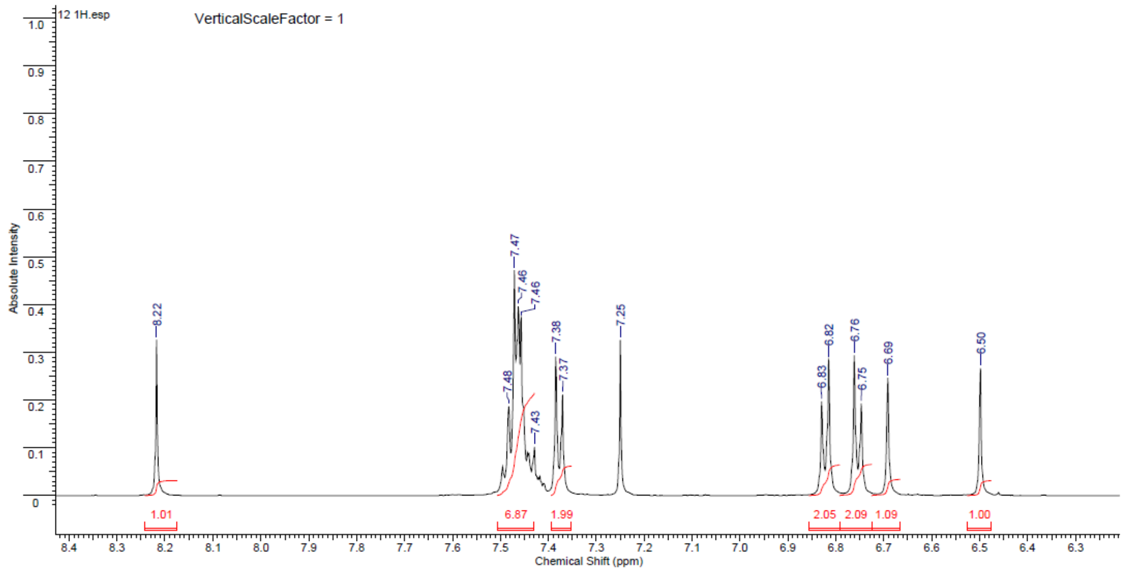

^{13}C NMR (150 MHz, $CDCl_3$)

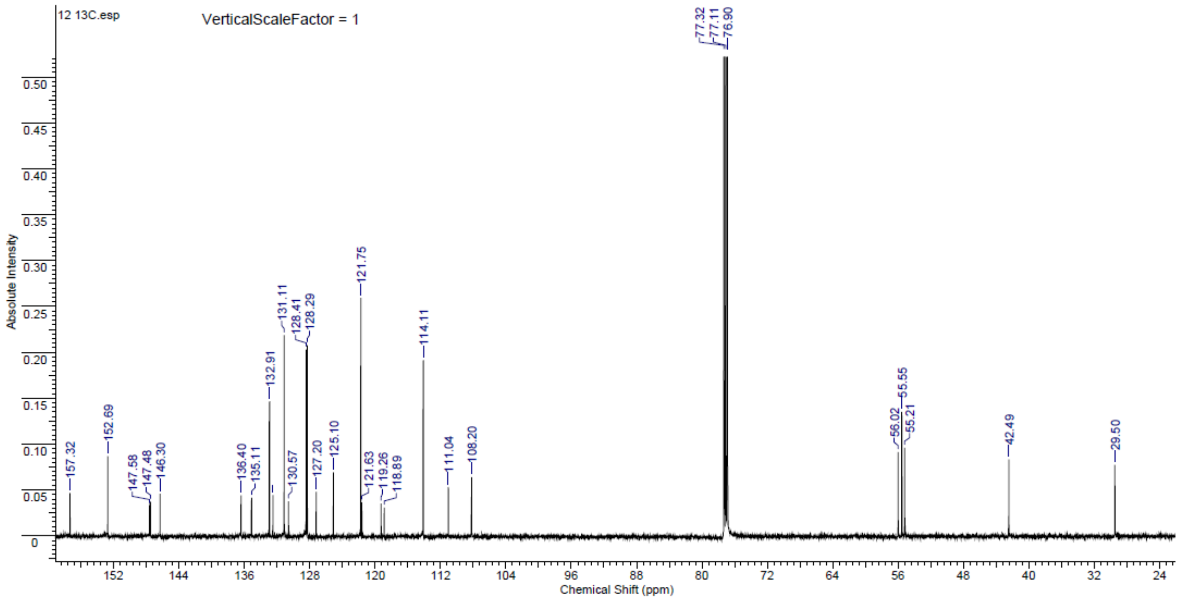

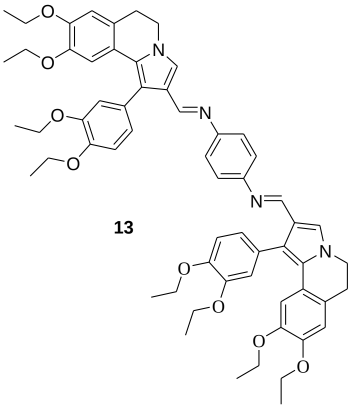

13

(1*E*,1'*E*)-*N*,*N'*-(1,4-phenylene)bis(1-(1-(3,4-diethoxyphenyl)-8,9-diethoxy-5,6-dihydropyrrolo[2,1-*a*]isoquinolin-2-yl)methanimine)

^{1}H NMR (400 MHz, DMSO-d_6)

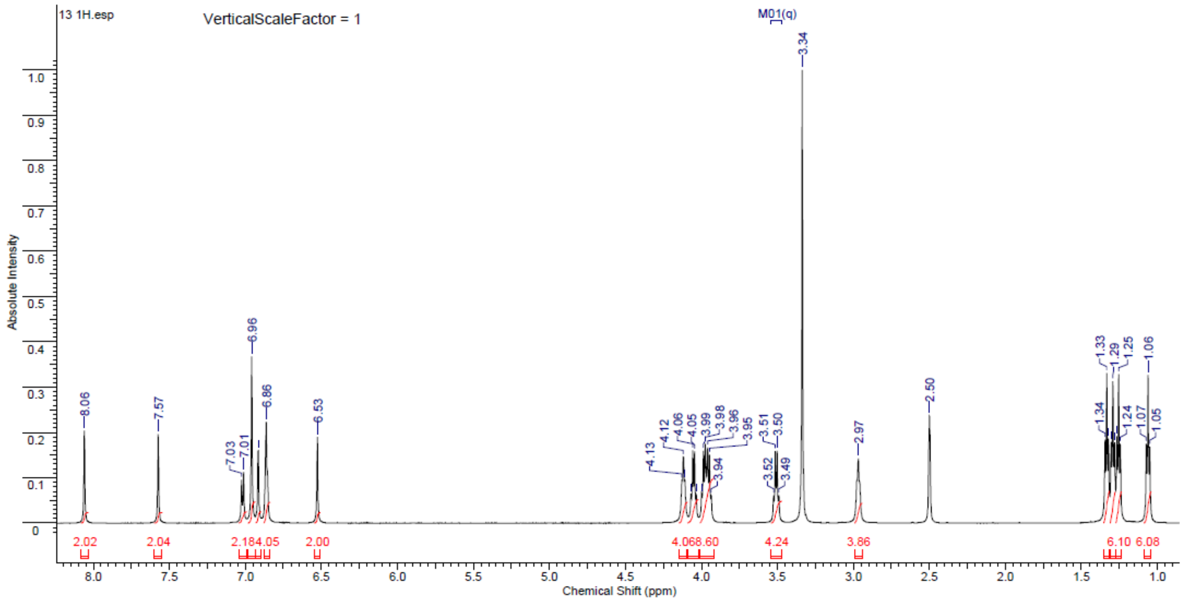

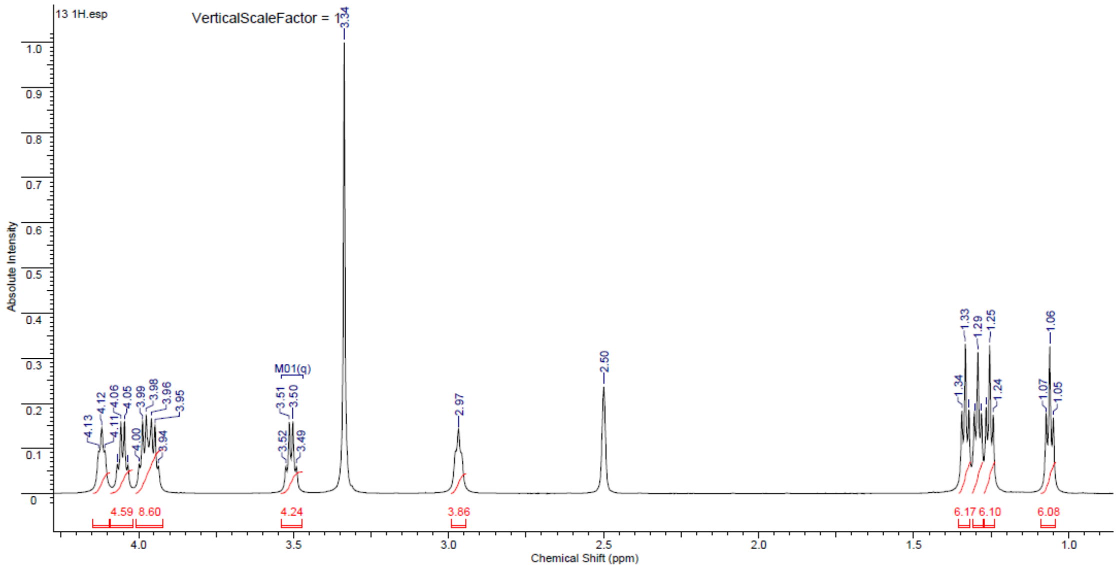

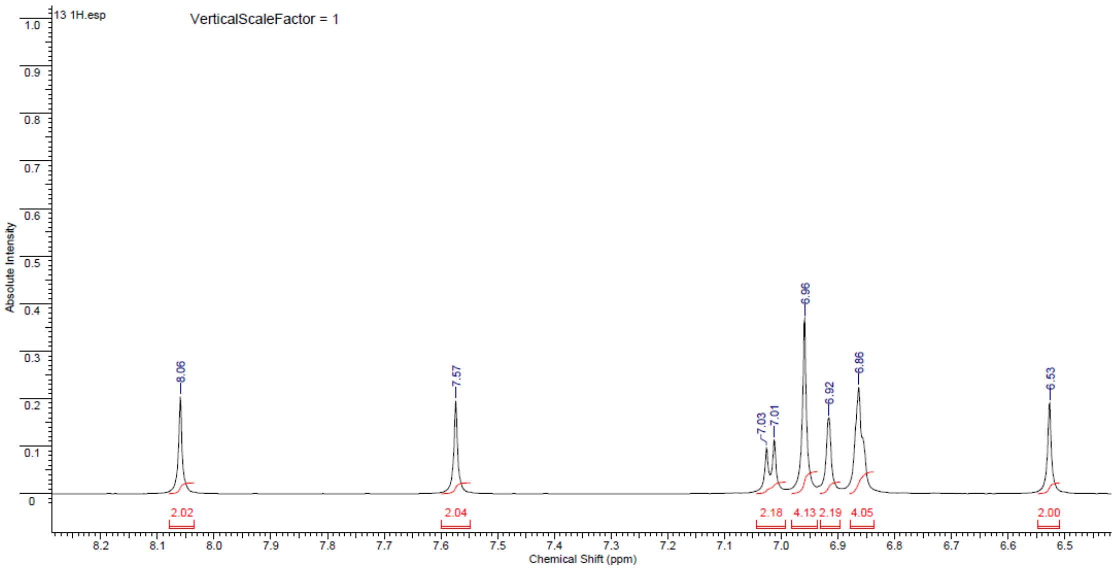

^{13}C NMR (150 MHz, DMSO-d_6)

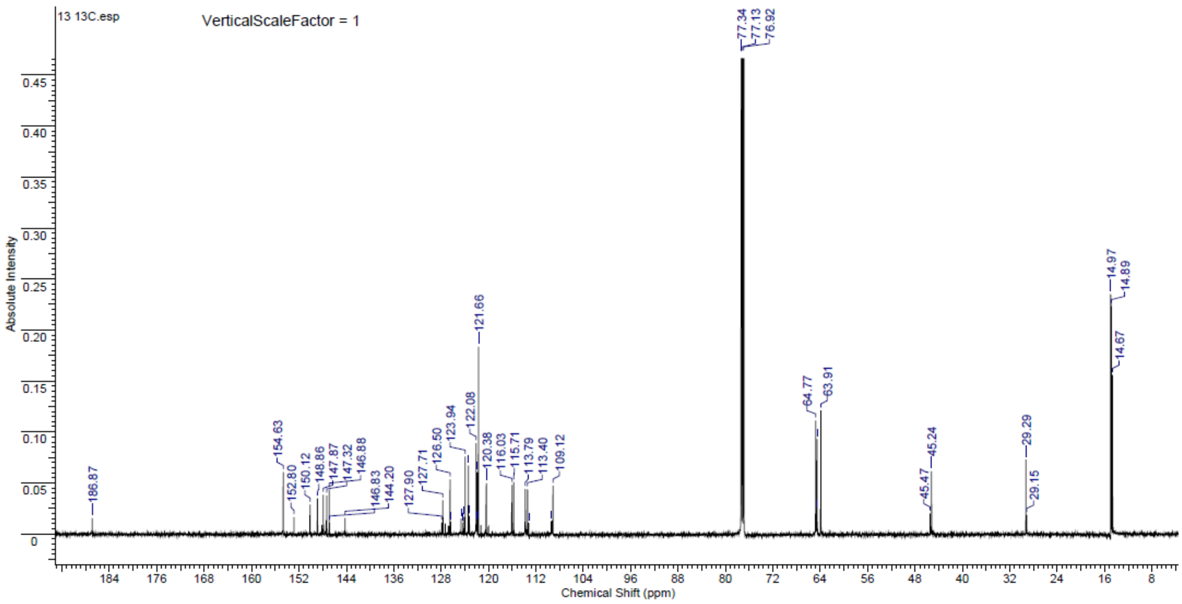

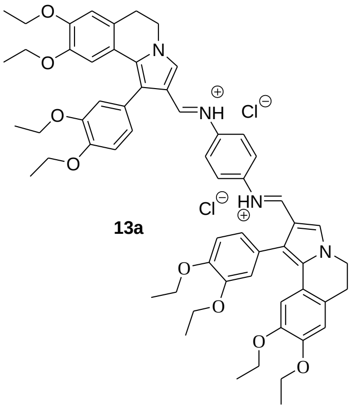

13a

(1*E*,1'*E*)-*N*,*N'*-(1,4-phenylene)bis(1-(1-(3,4-diethoxyphenyl)-8,9-diethoxy-5,6-dihydropyrrolo[2,1-*a*]isoquinolin-2-yl)methanimine)-1,4-diaminium dichloride

^{1}H NMR (600 MHz, $CDCl_3$)

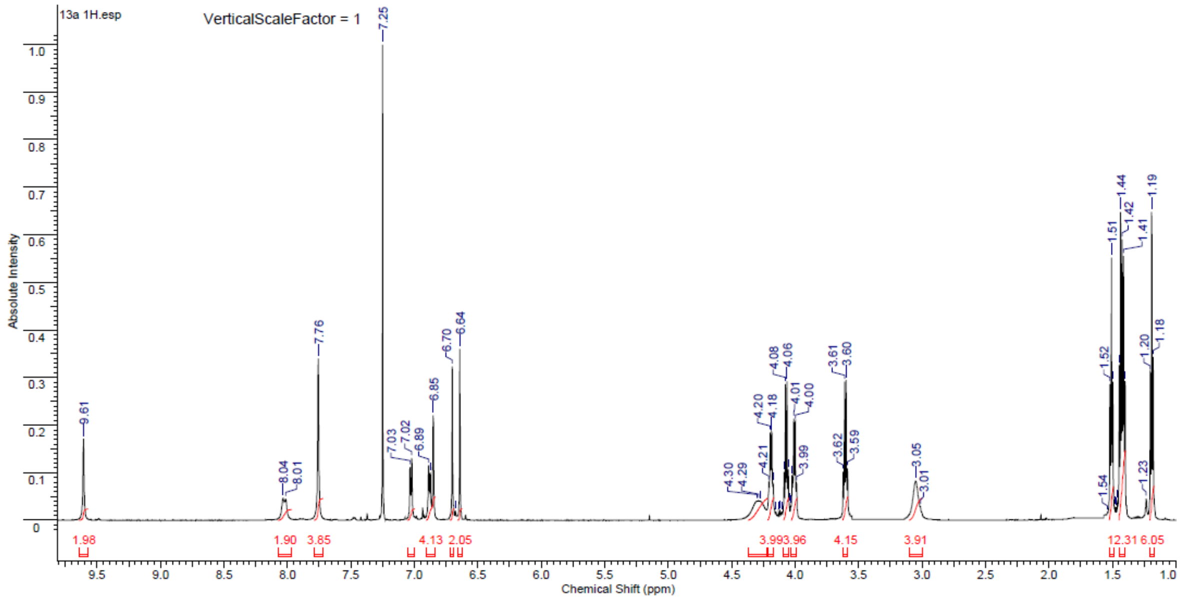

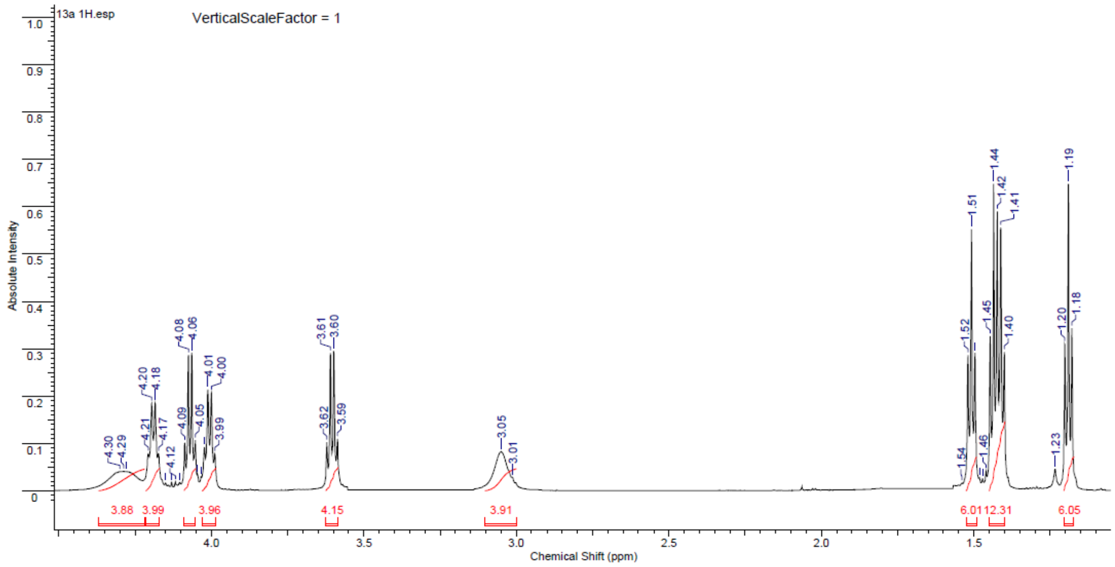

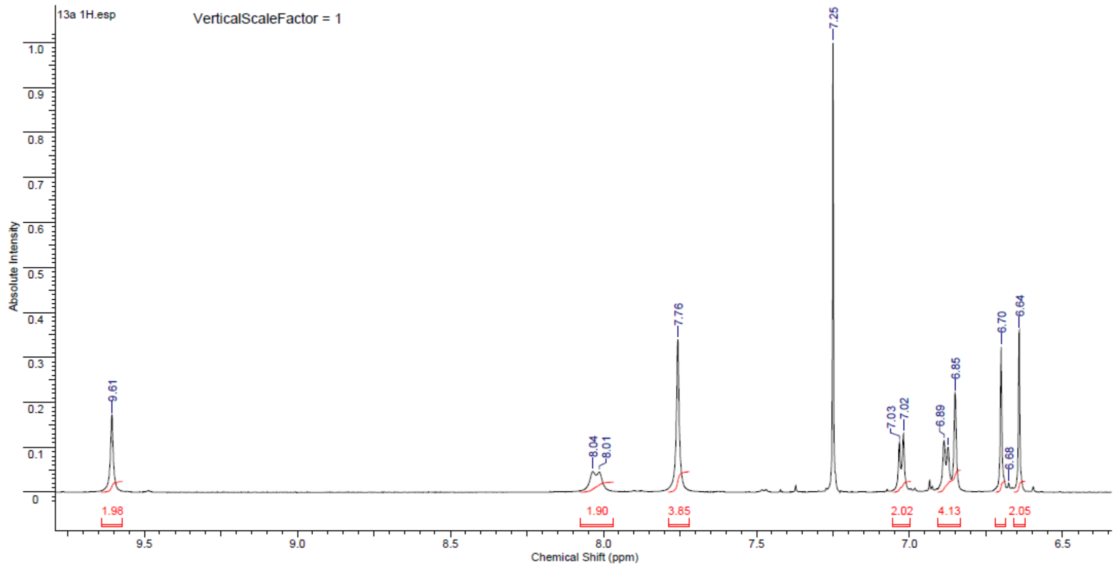

^{13}C NMR (150 MHz, DMSO-d_6)

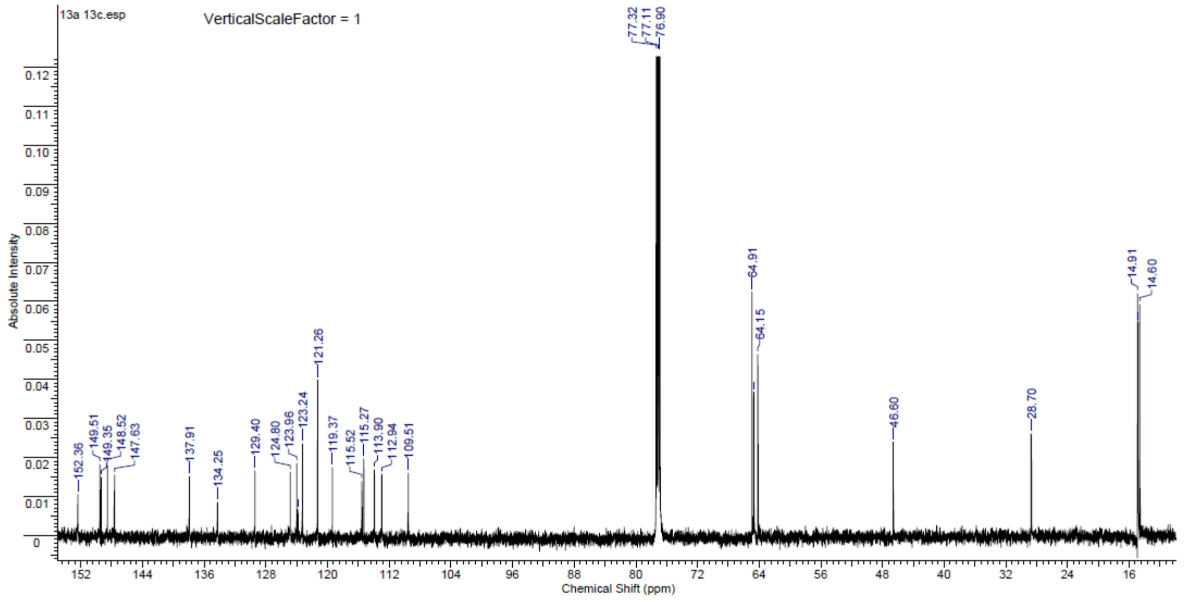

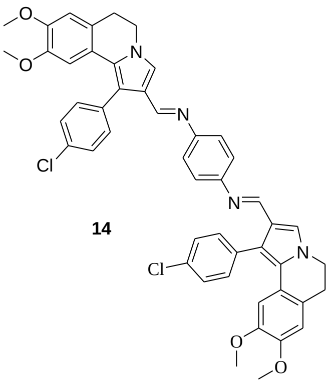

14

(1*E*,1'*E*)-*N*,*N*'-(1,4-phenylene)bis(1-(1-(4-chlorophenyl)-8,9-dimethoxy-5,6-dihydropyrrolo[2,1-*a*]isoquinolin-2-yl)methanimine)

^{1}H NMR (600 MHz, $CDCl_3$)

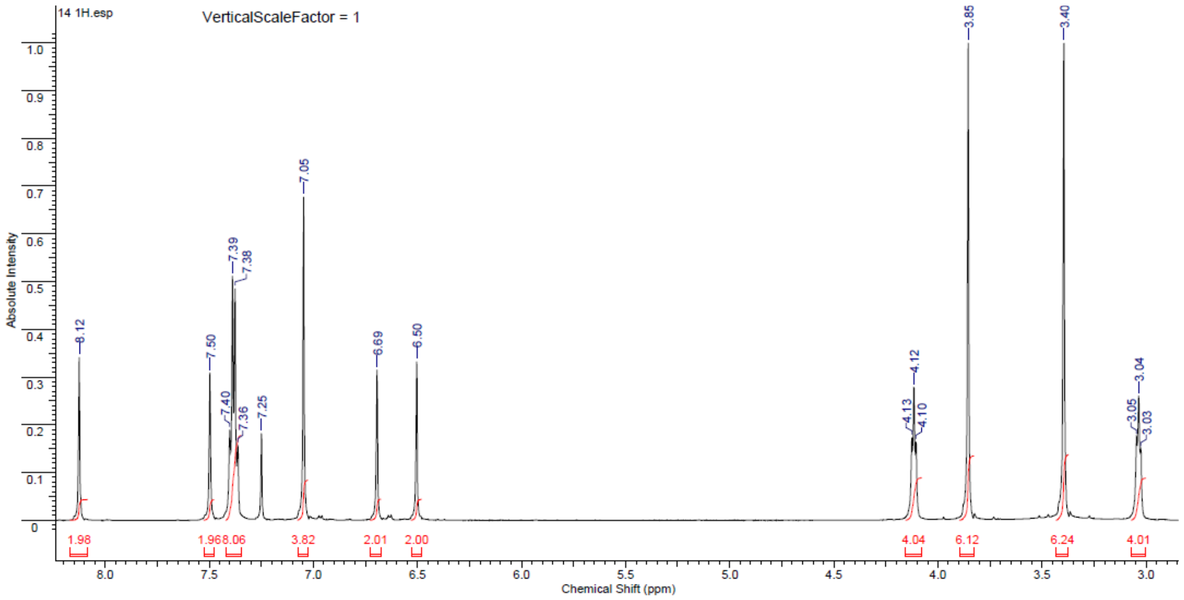

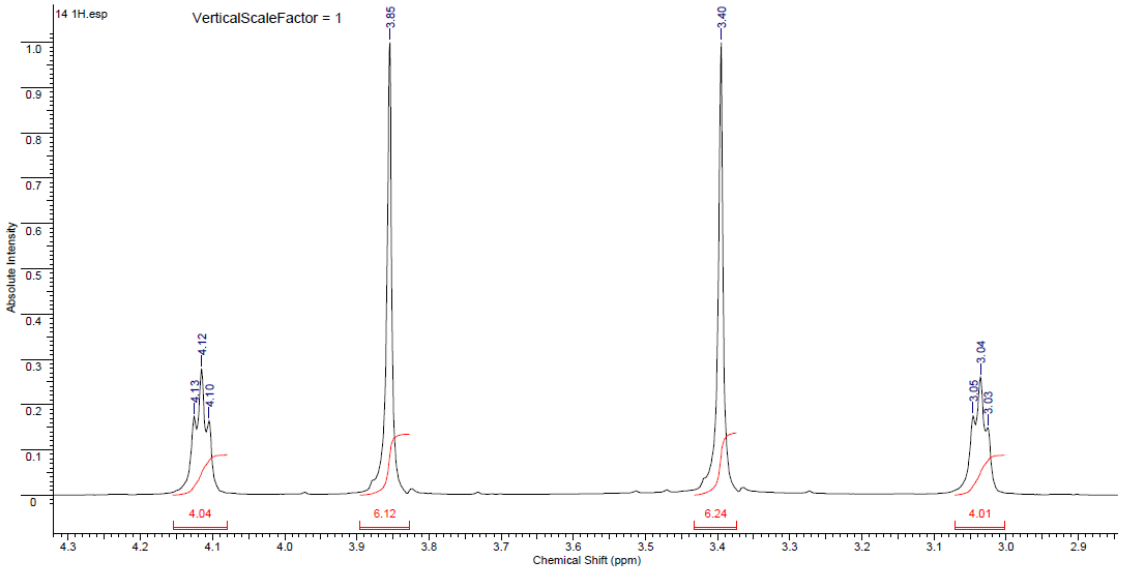

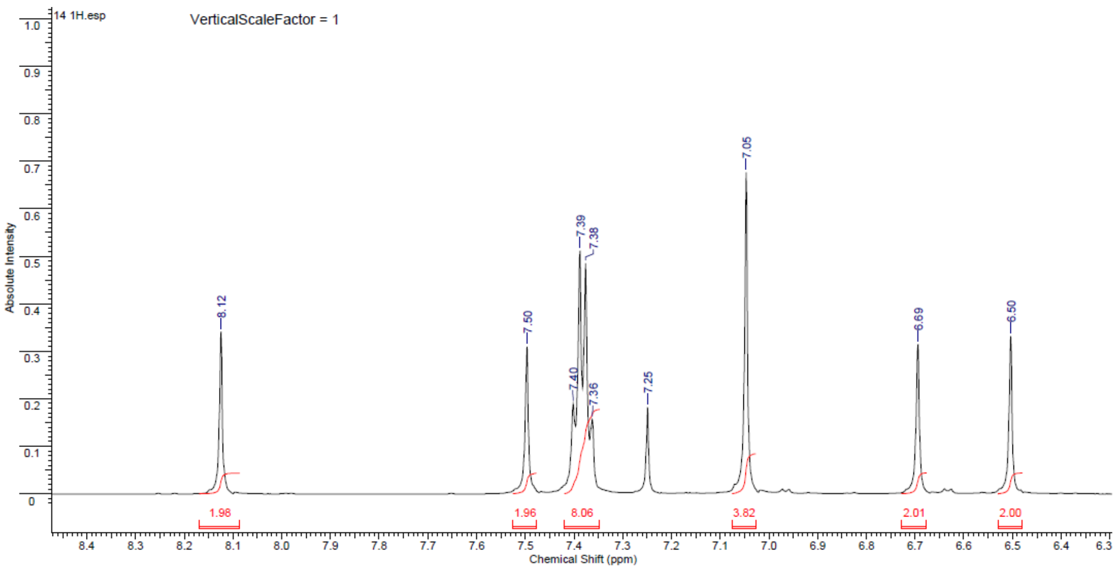

^{13}C NMR (150 MHz, DMSO-d_6)

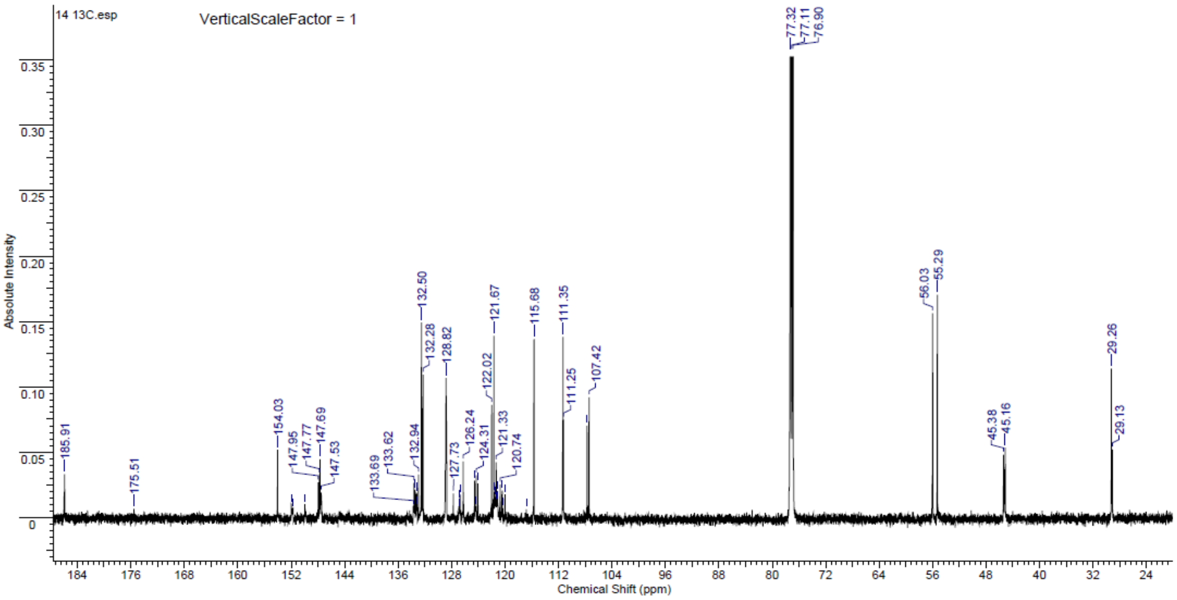

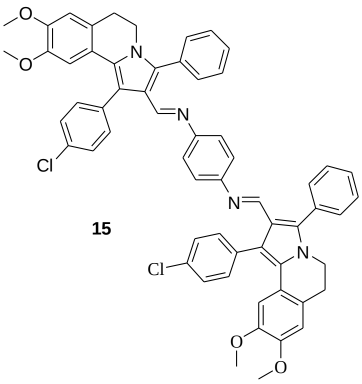

15

(1*E*,1'*E*)-*N*,*N'*-(1,4-phenylene)bis(1-(1-(4-chlorophenyl)-8,9-dimethoxy-3-phenyl-5,6-dihydropyrrolo[2,1-*a*]isoquinolin-2-yl)methanimine)

^{1}H NMR (400 MHz, DMSO-d_6)

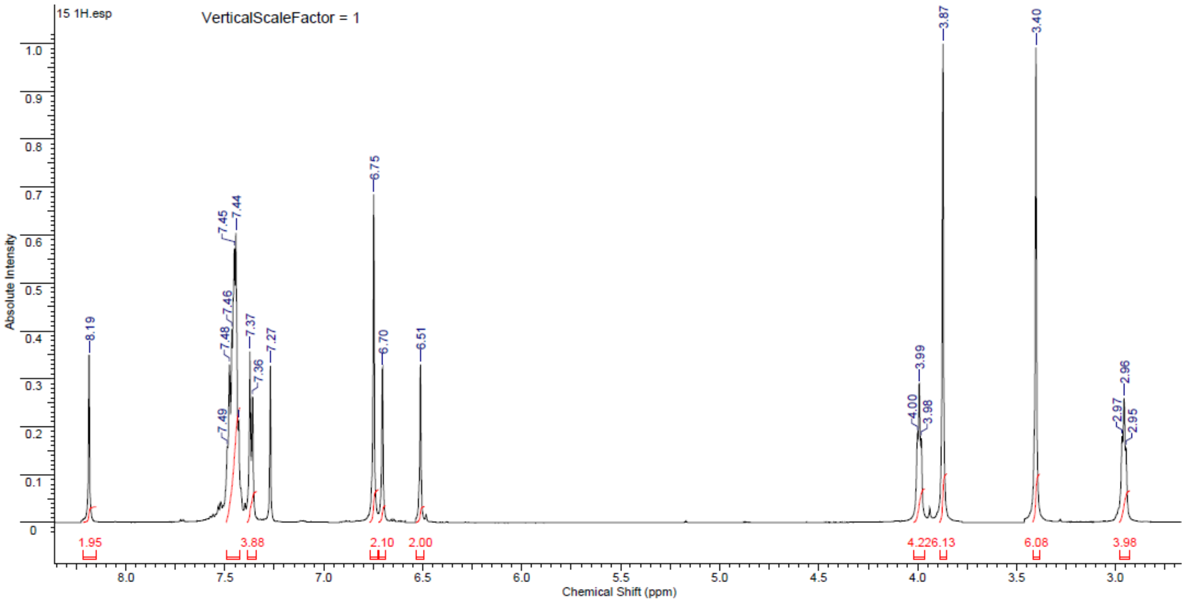

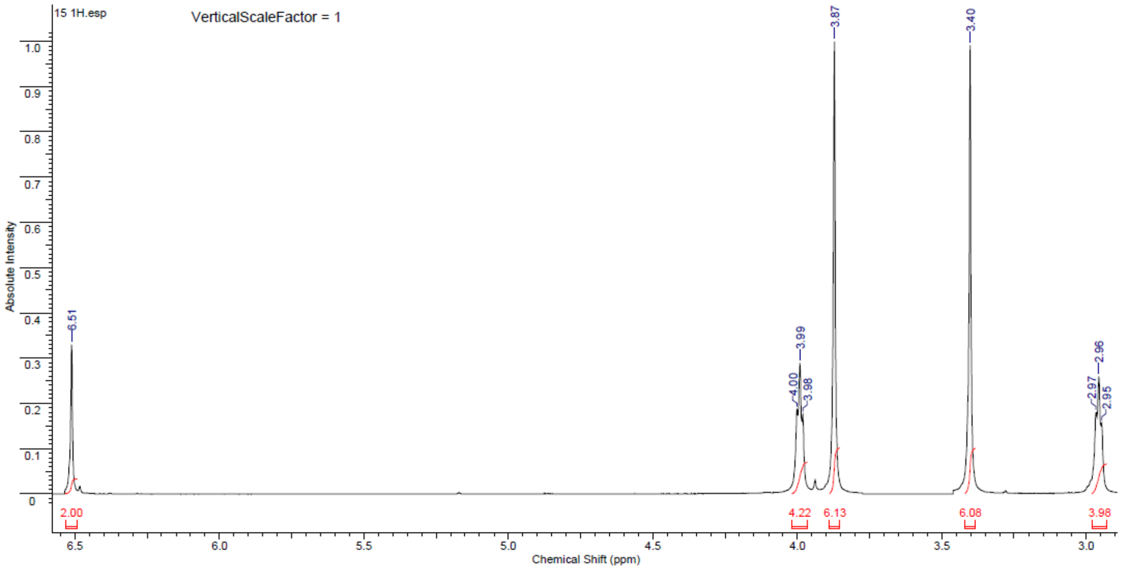

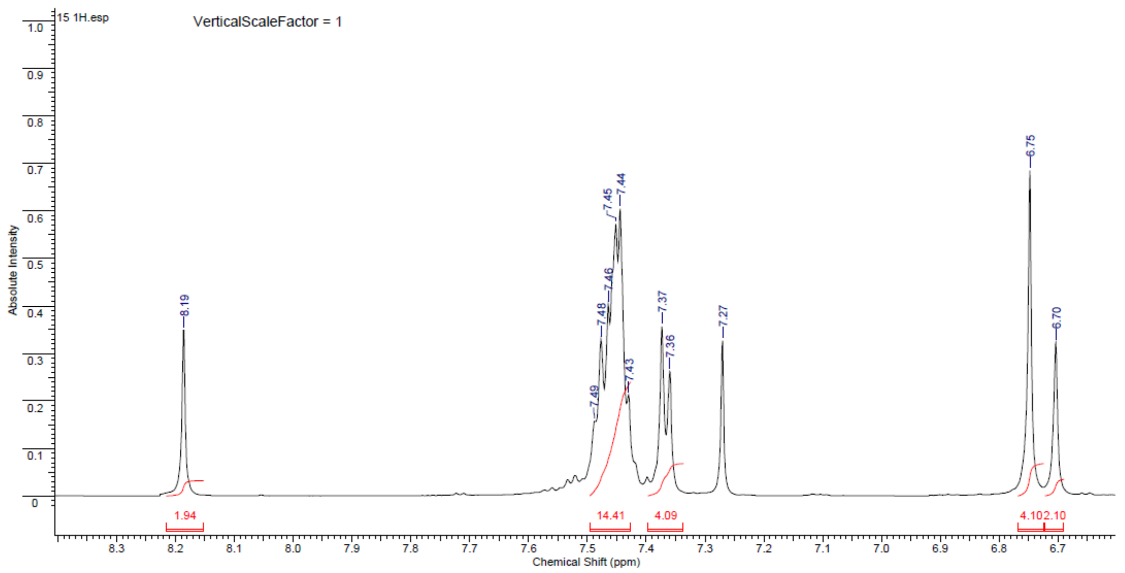

^{13}C NMR (150 MHz, DMSO-d_6)

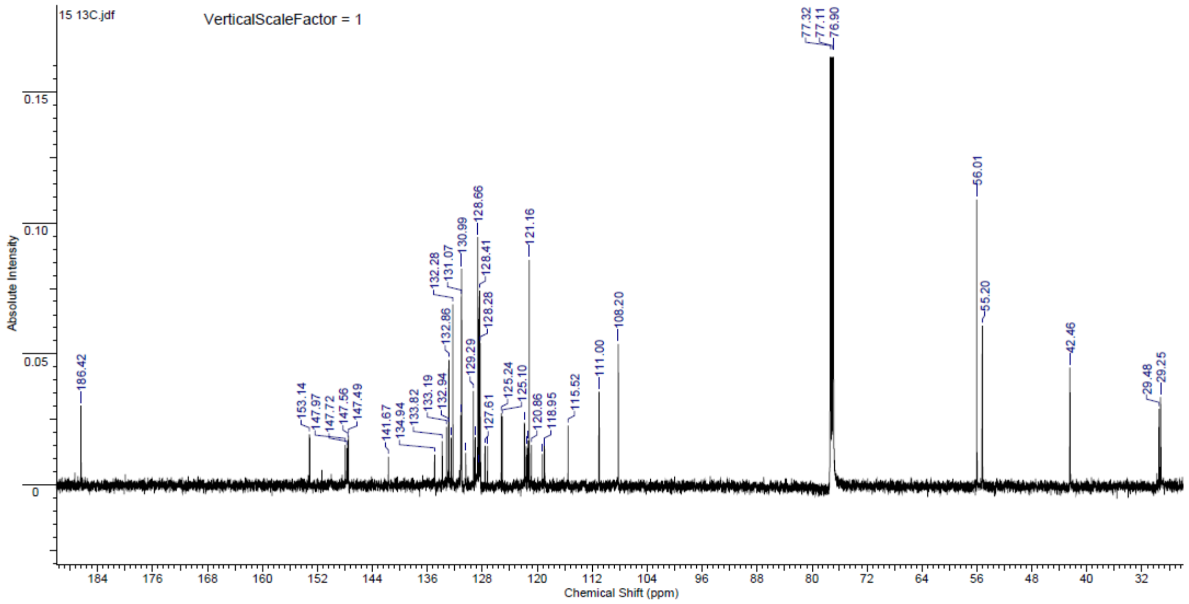

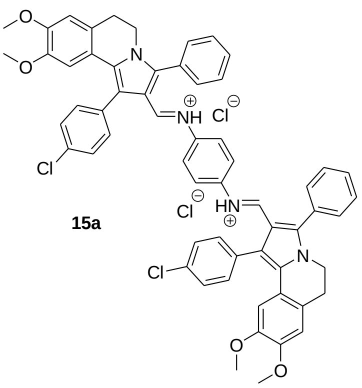

15a

(1*E*,1'*E*)-*N*,*N*'-(1,4-phenylene)bis(1-(1-(3,4-diethoxyphenyl)-8,9-diethoxy-5,6-dihydropyrrolo[2,1-*a*]isoquinolin-2-yl)methanimine)-1,4-diaminium dichloride

^{1}H NMR (400 MHz, DMSO-d_6)

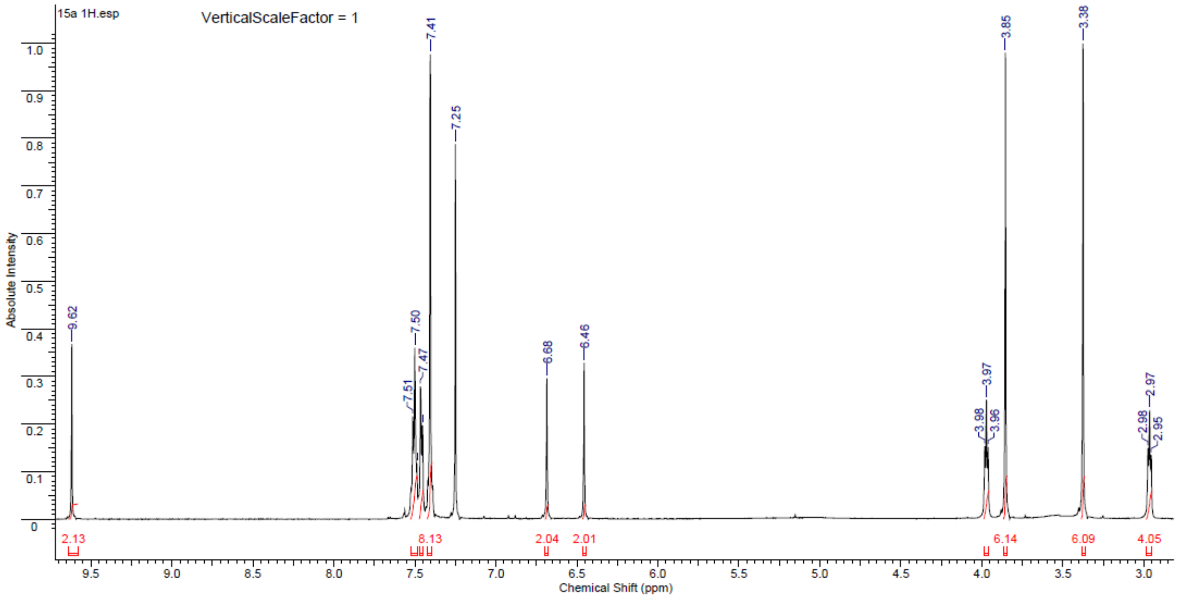

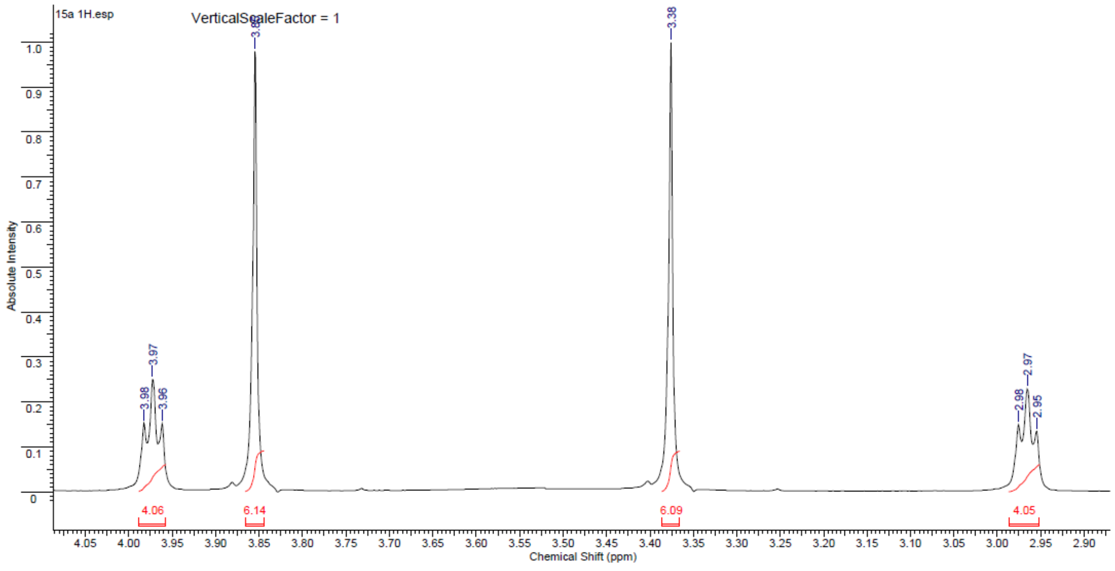

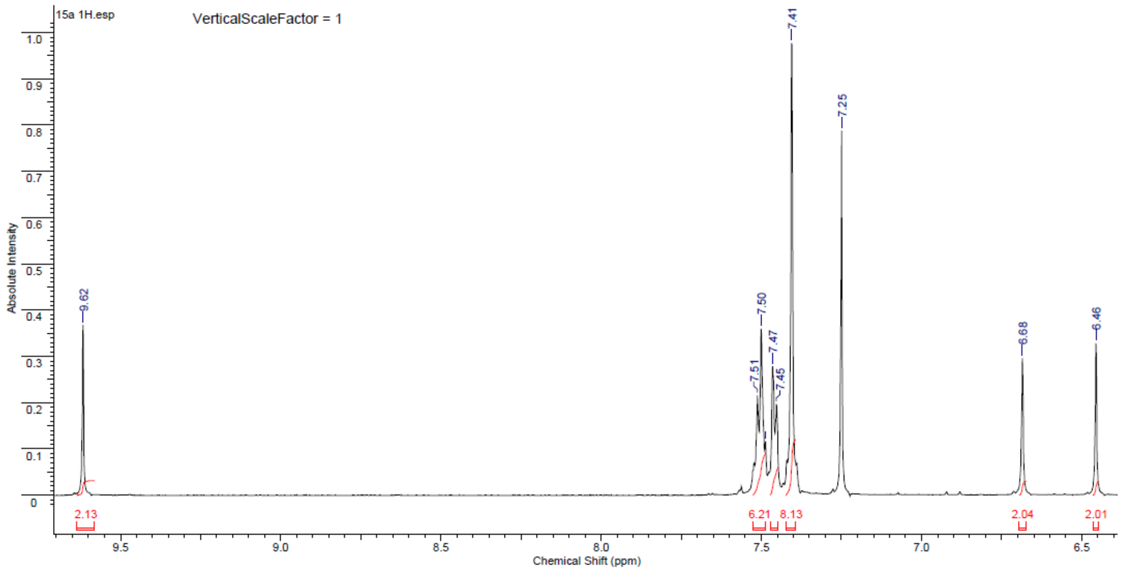

^{13}C NMR (150 MHz, DMSO-d_6)

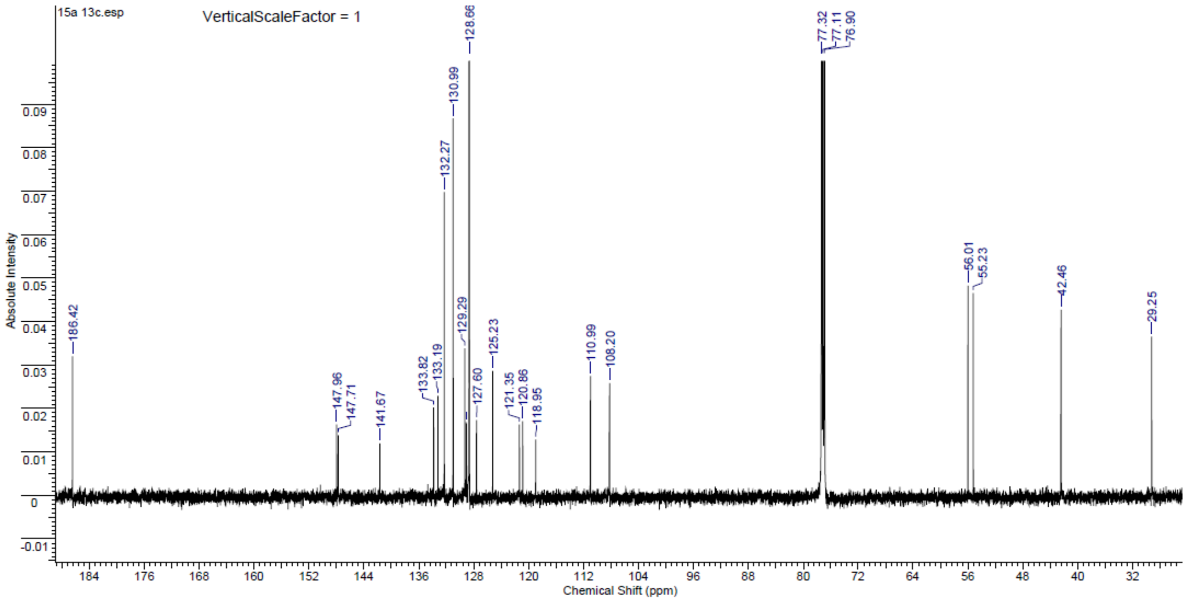

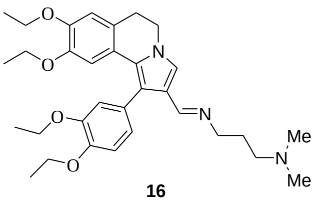

16

(*E*)-3-(((1-(3,4-diethoxyphenyl)-8,9-diethoxy-5,6-dihydropyrrolo[2,1-*a*]isoquinolin-2-yl)methylene)amino)-*N*,*N*-dimethylpropan-1-amine

^{1}H NMR (600 MHz, $CDCl_3$)

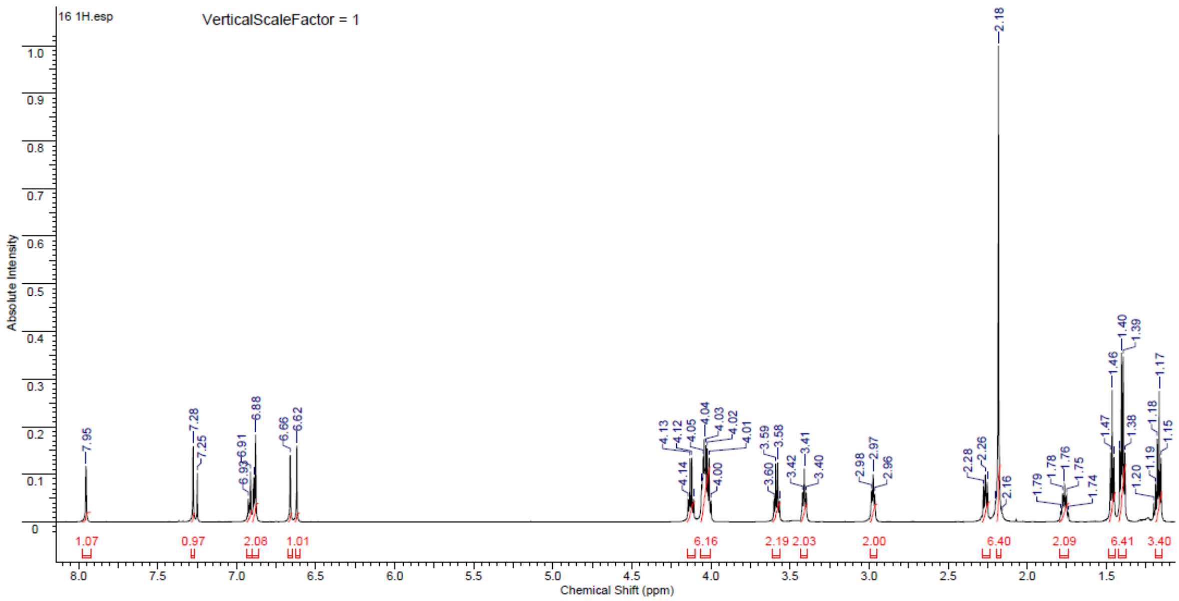

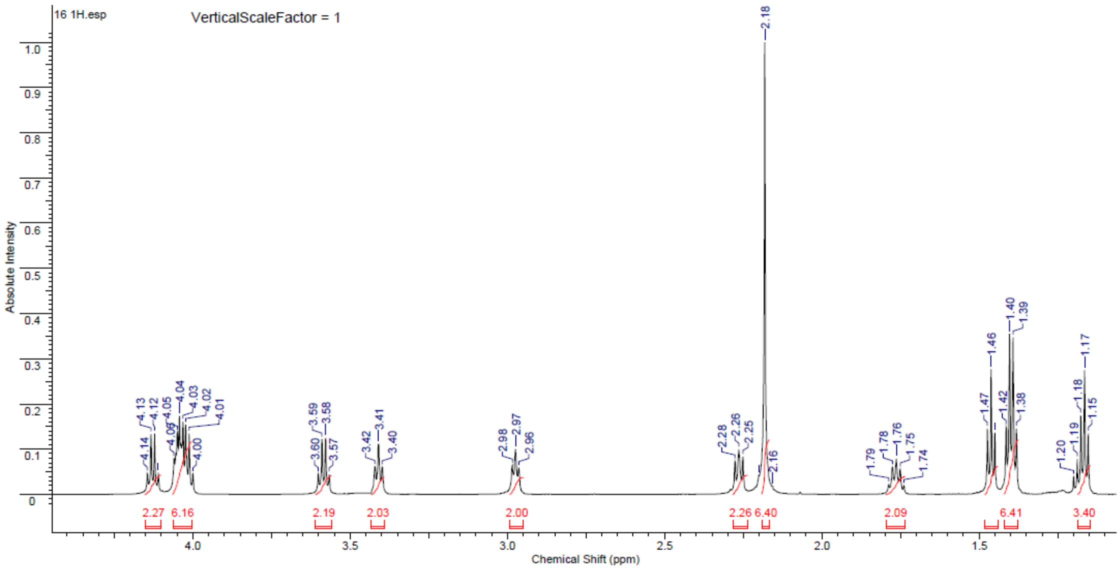

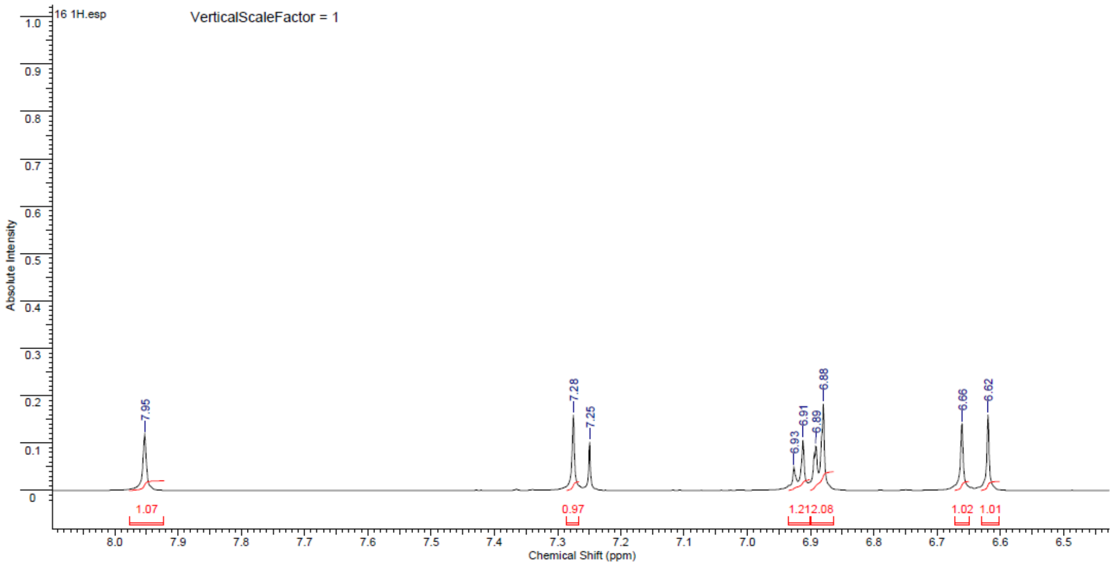

^{13}C NMR (150 MHz, DMSO-d_6)

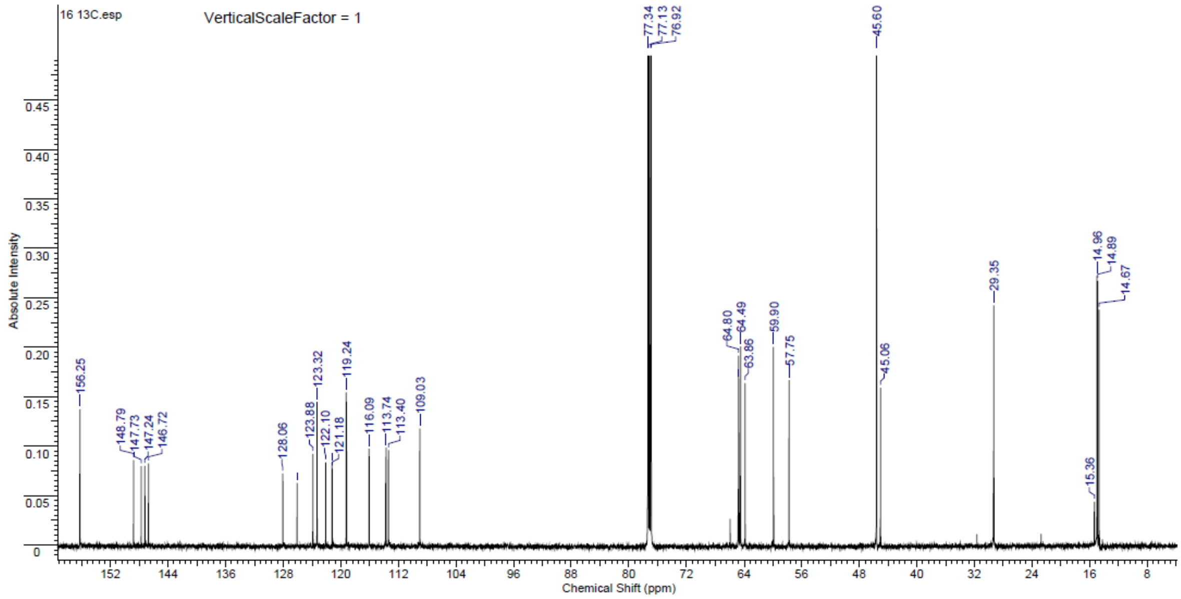

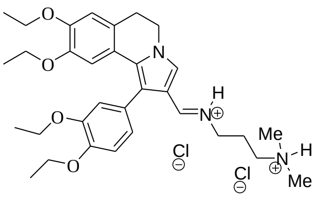

16a

(*E*)-N^1-((1-(3,4-diethoxyphenyl)-8,9-diethoxy-5,6-dihydropyrrolo[2,1-*a*]isoquinolin-2-yl)methylene)-N^3,N^3-dimethylpropane-1,3-diaminium chloride

^{1}H NMR (600 MHz, DMSO-d_6)

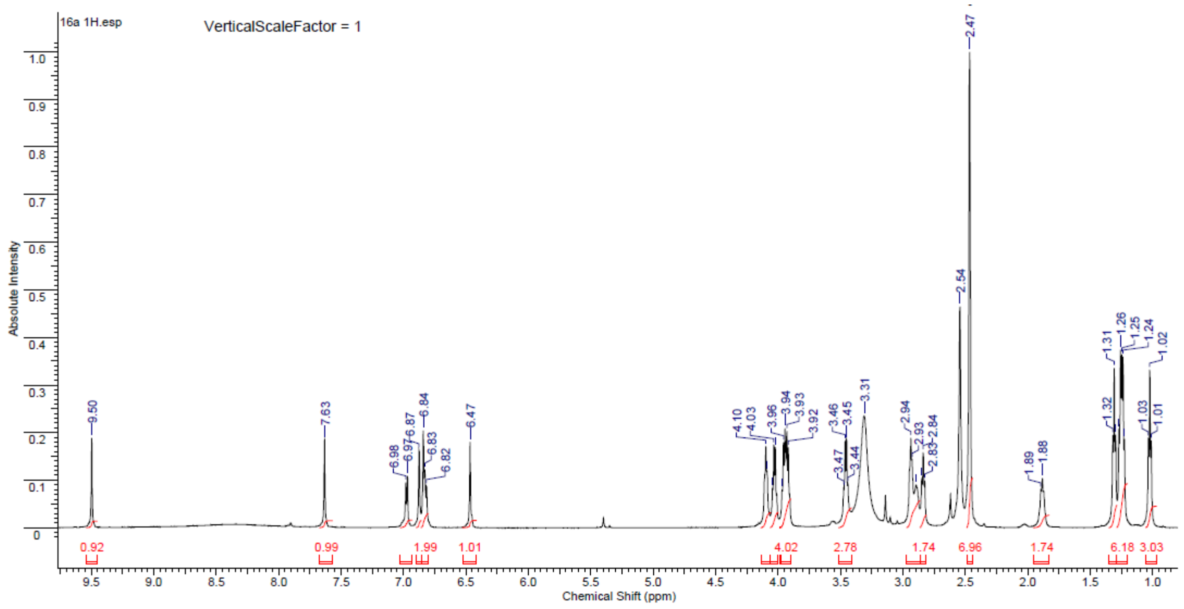

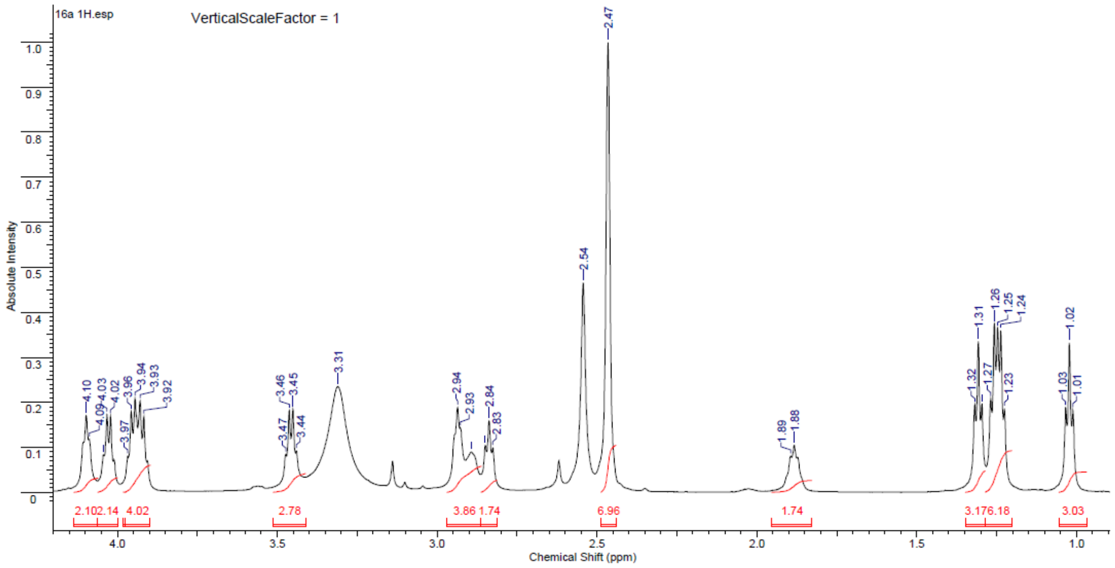

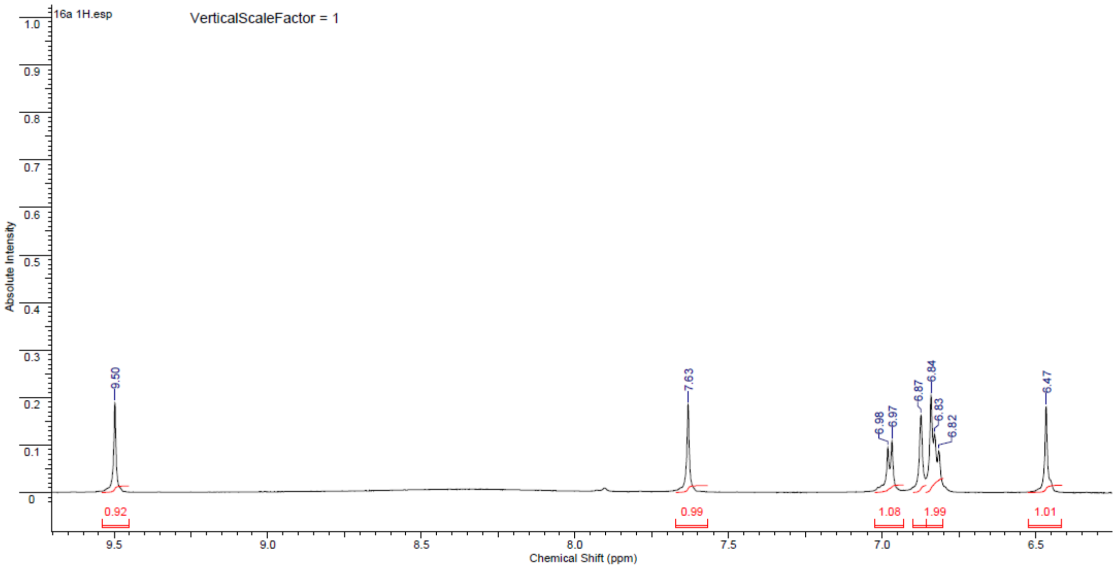

^{13}C NMR (150 MHz, DMSO-d_6)

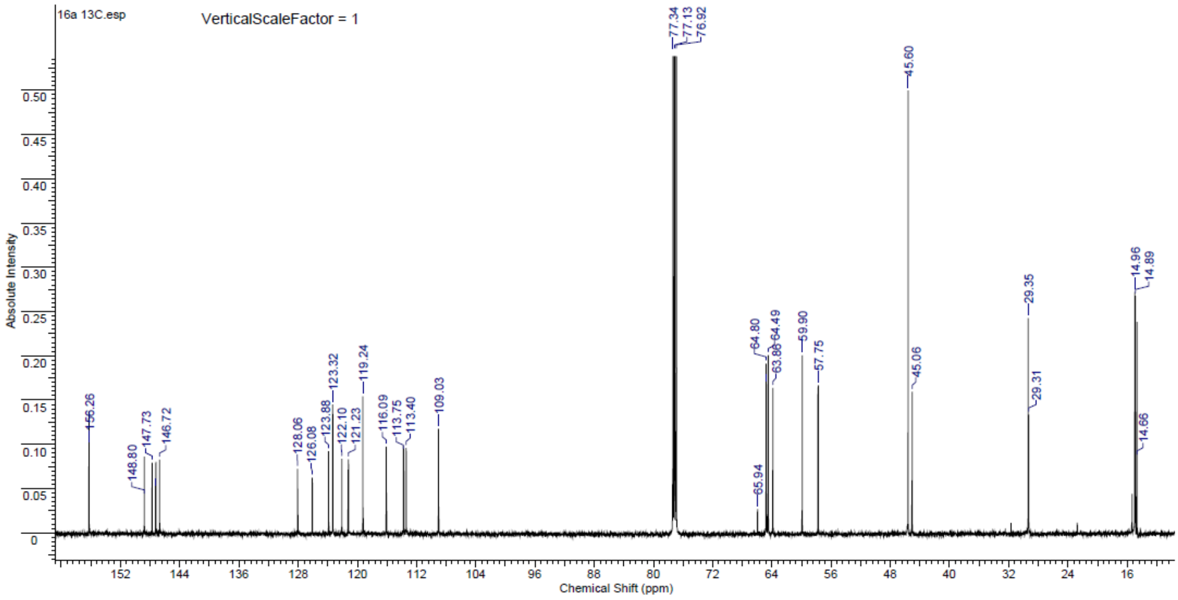

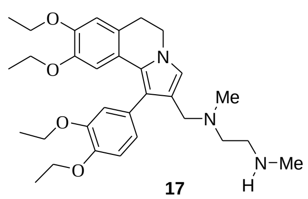

17

N[1]-((1-(3,4-diethoxyphenyl)-8,9-diethoxy-5,6-dihydropyrrolo[2,1-*a*]isoquinolin-2-yl)methyl)-*N*[1],*N*[2]-dimethylethane-1,2-diamine

^{1}H NMR (600 MHz, $CDCl_3$)

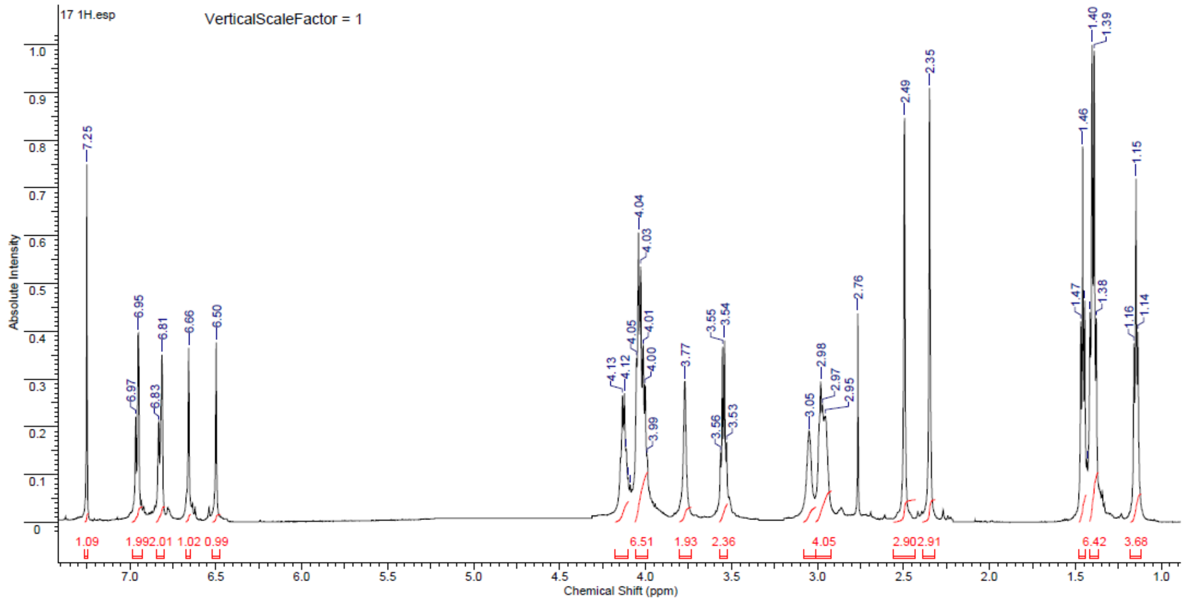

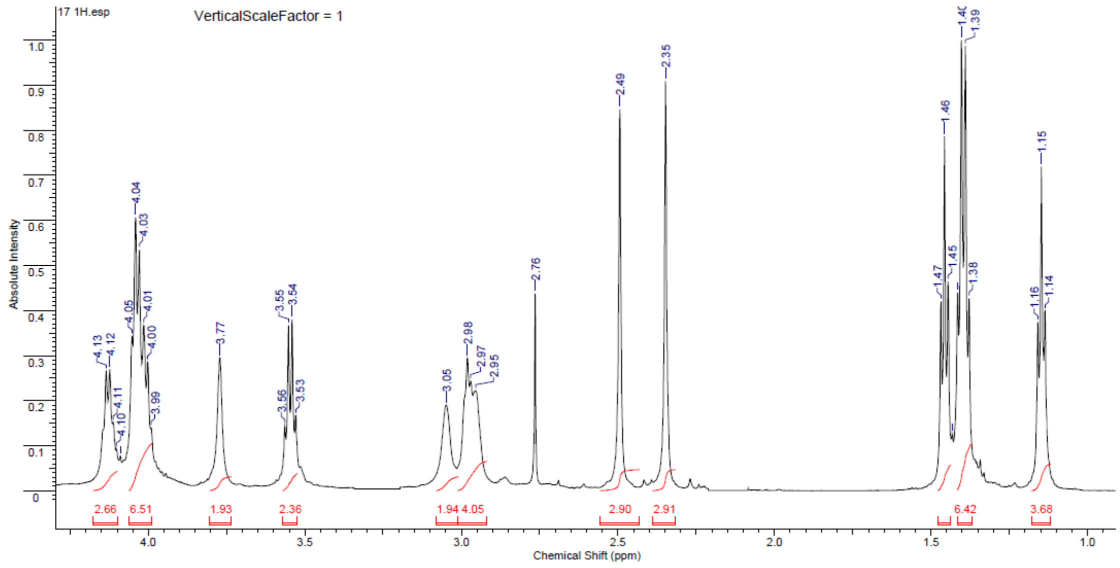

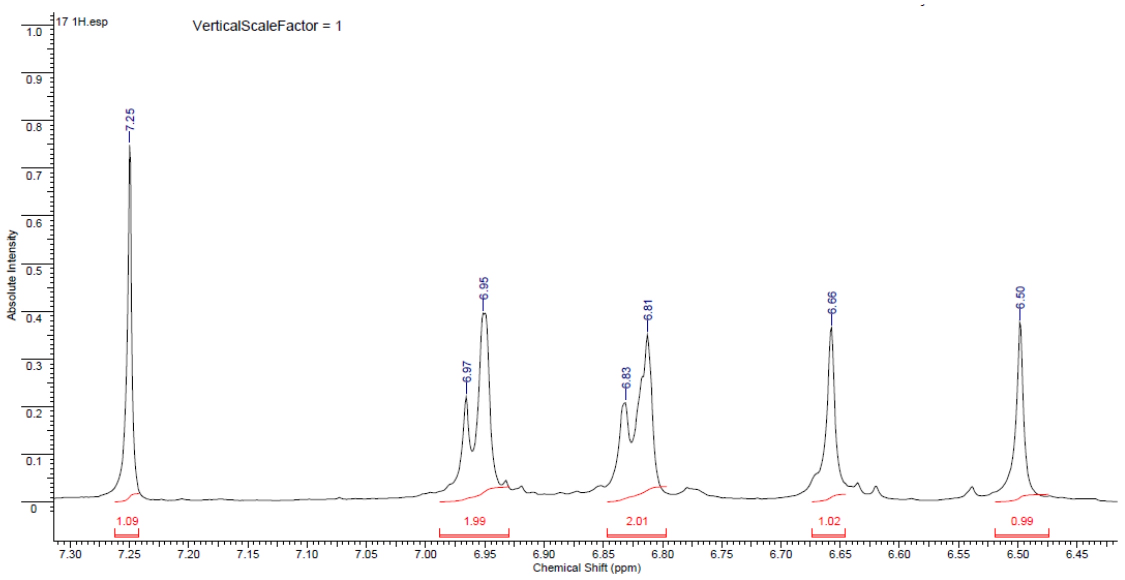

^{13}C NMR (150 MHz, DMSO-d_6)

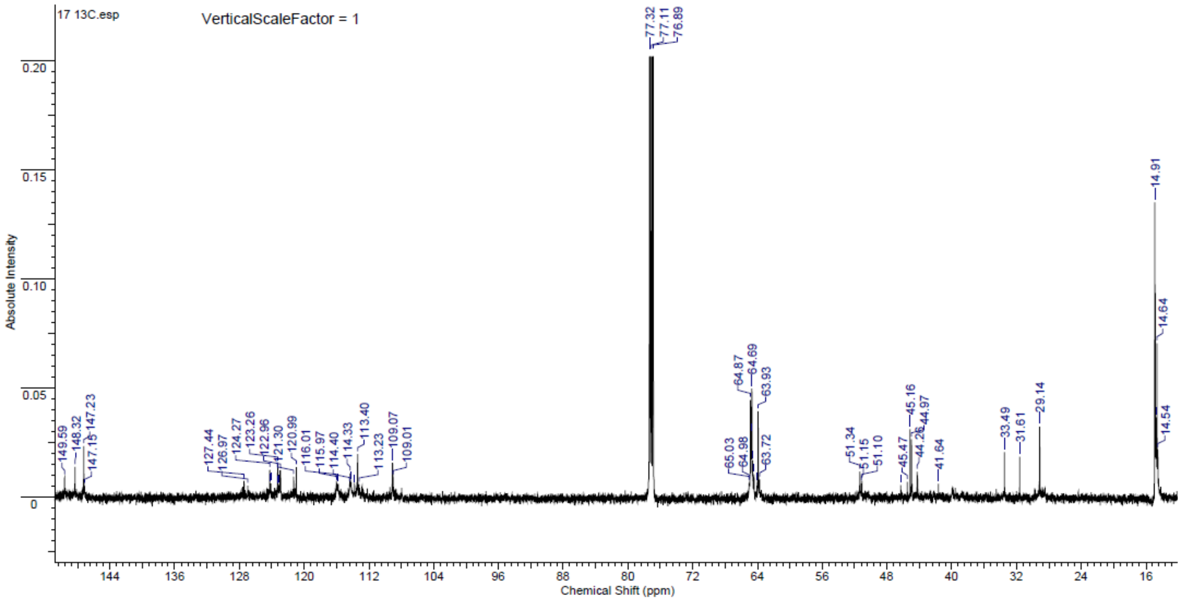

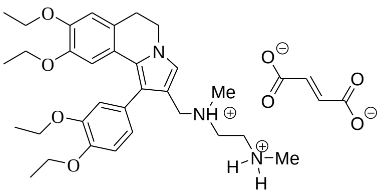

17a

2-(((1-(3,4-diethoxyphenyl)-8,9-diethoxy-5,6-dihydropyrrolo[2,1-*a*]isoquinolin-2-yl)methyl)(methyl)amino)-*N*-methylethan-1-aminium (*E*)-3-carboxyacrylate

^{1}H NMR (600 MHz, DMSO-d_6)

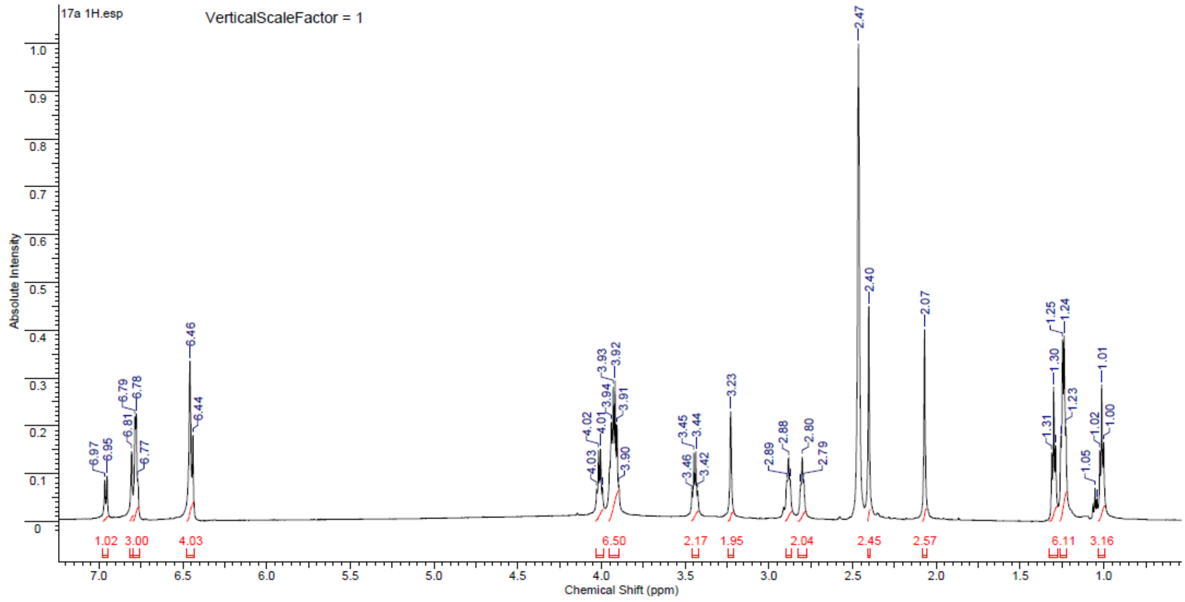

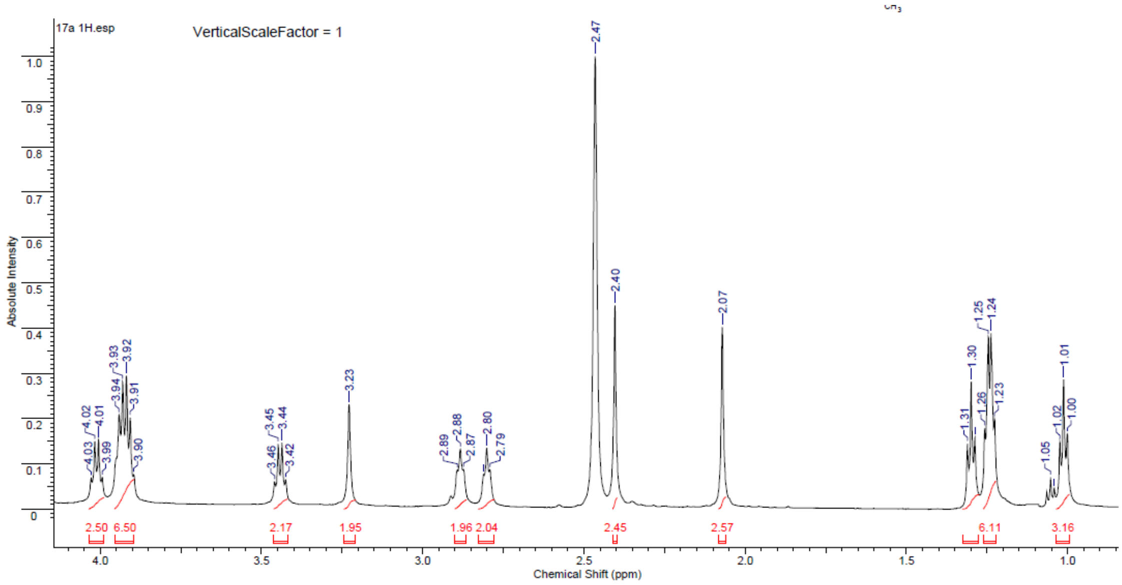

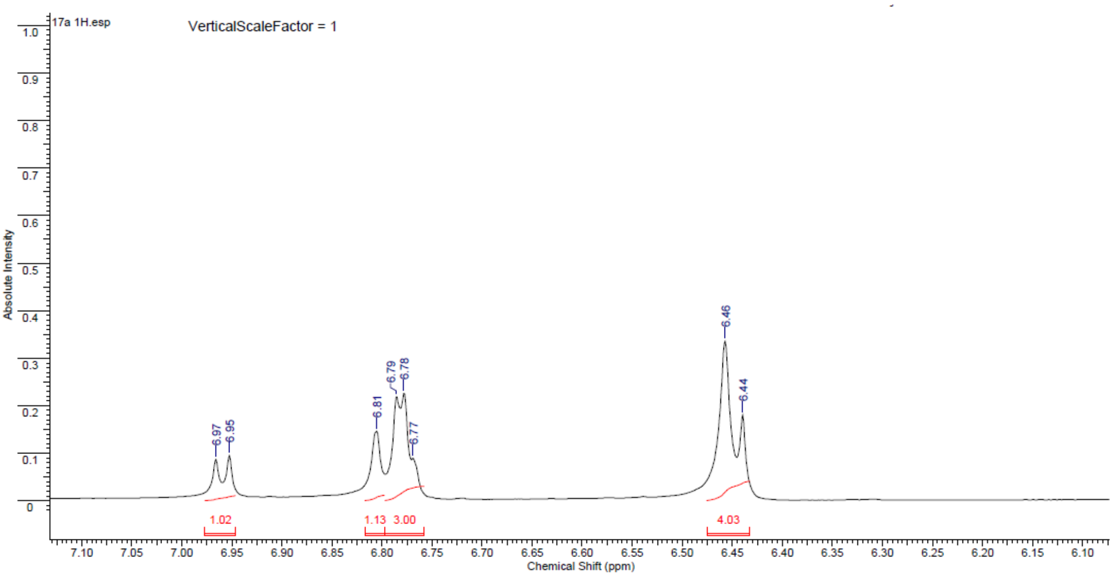

^{13}C NMR (150 MHz, DMSO-d_6)

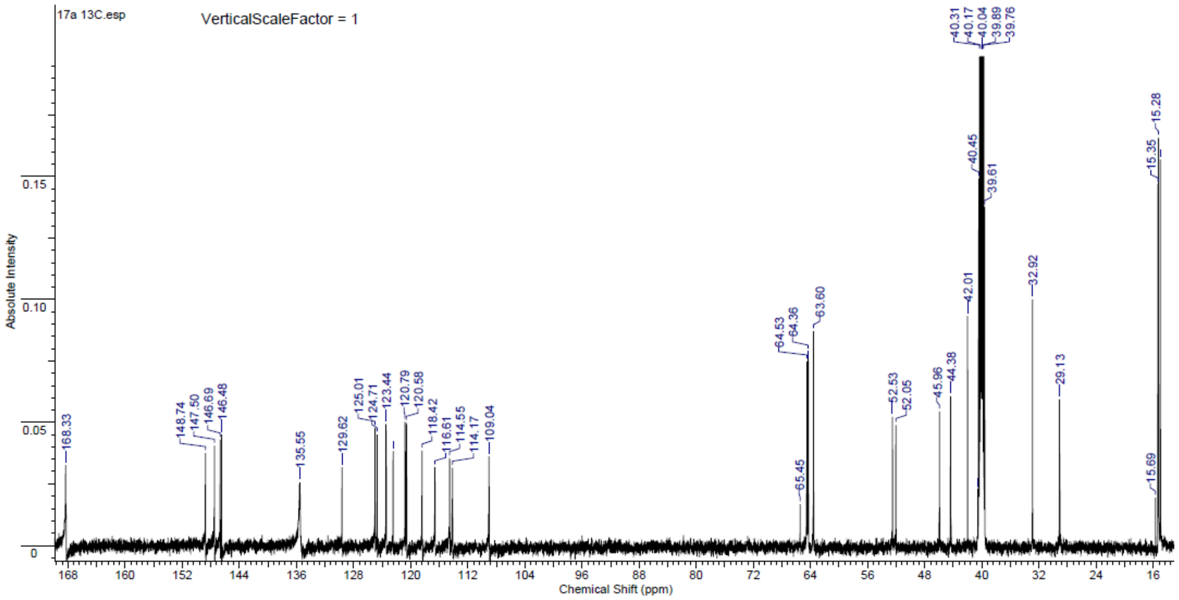

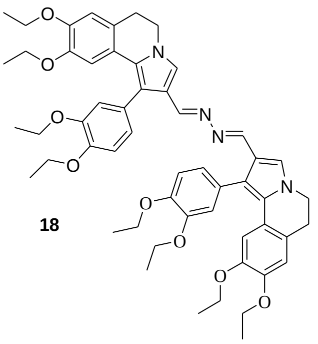

18

(1*E*,2*E*)-1,2-bis((1-(3,4-diethoxyphenyl)-8,9-diethoxy-5,6-dihydropyrrolo[2,1-*a*]isoquinolin-2-yl)methylene)hydrazine

^{1}H NMR (600 MHz, DMSO-d_6)

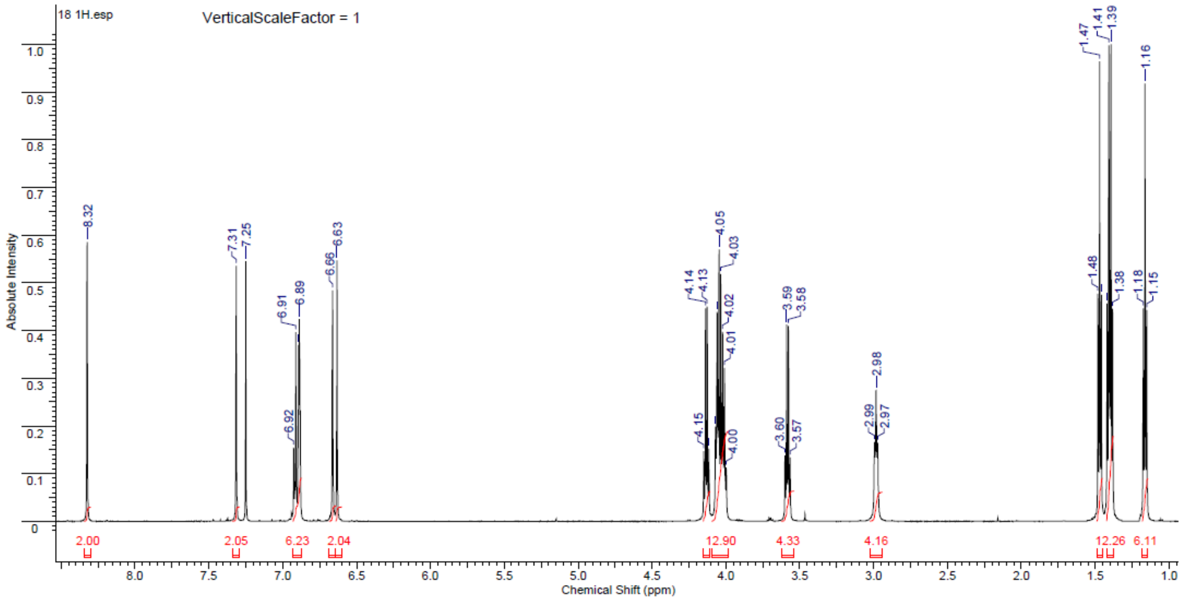

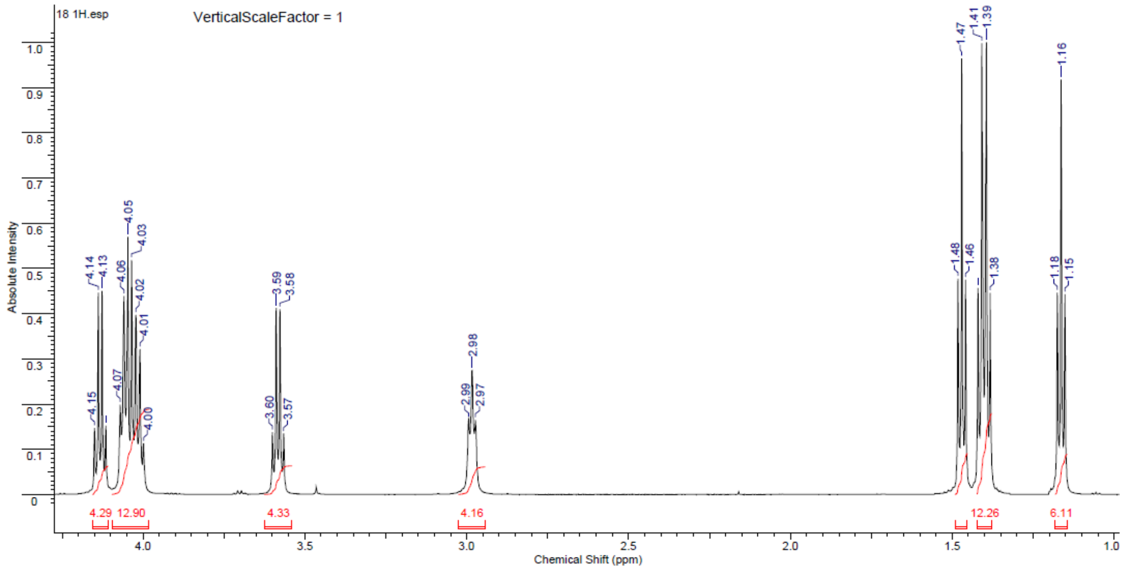

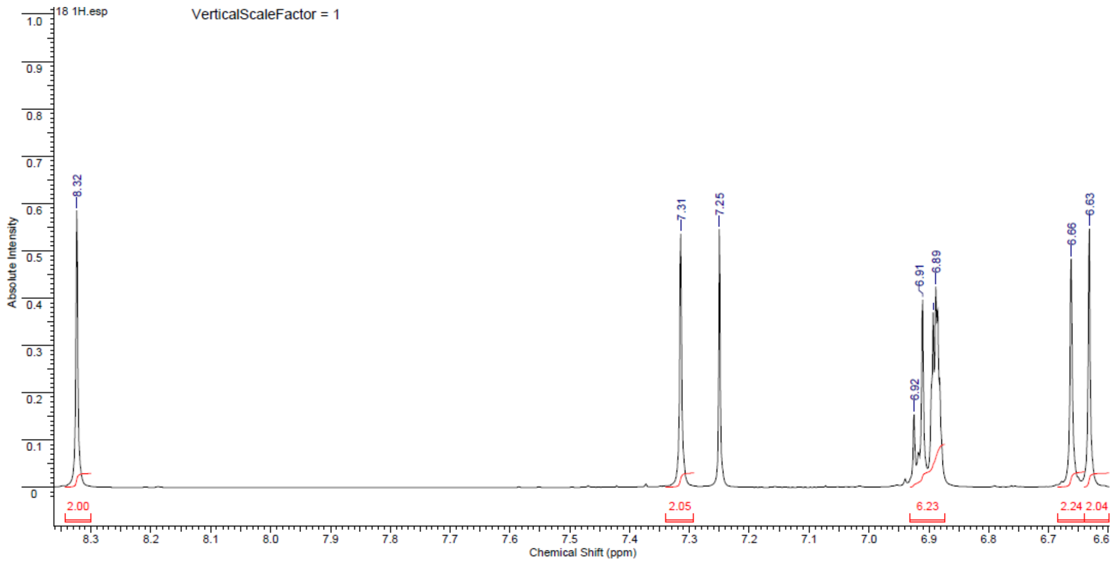

^{13}C NMR (150 MHz, DMSO-d_6)

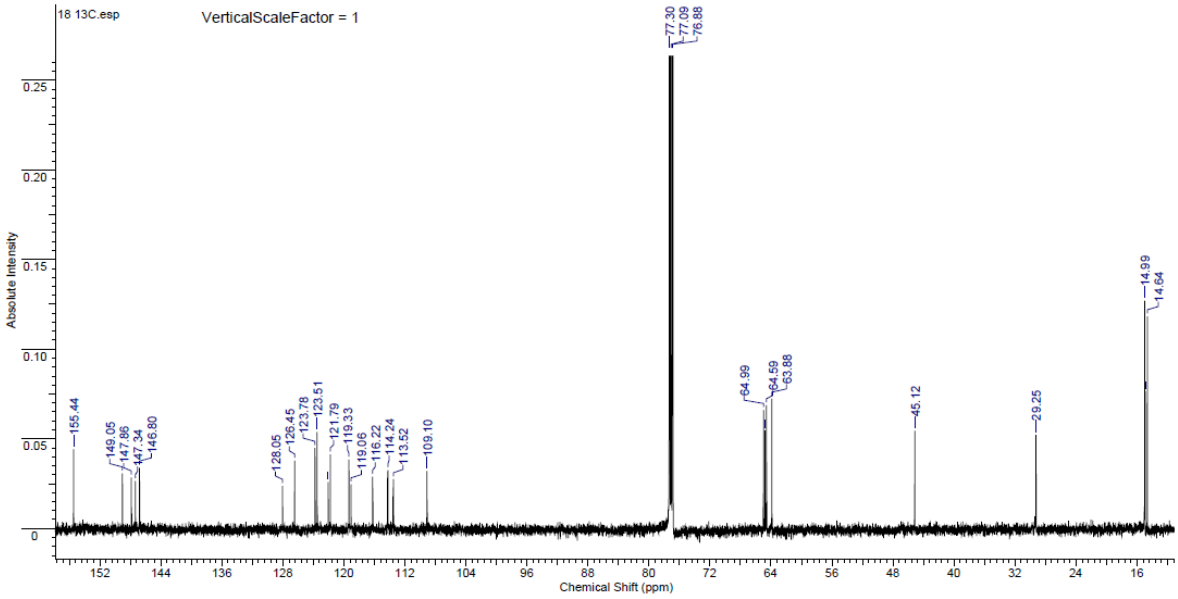

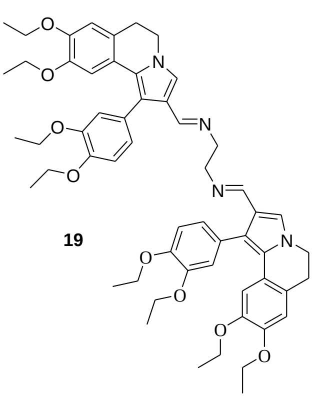

19

(1*E*,1'*E*)-*N*,N'-(ethane-1,2-diyl)bis(1-(1-(3,4-diethoxyphenyl)-8,9-diethoxy-5,6-dihydropyrrolo[2,1-*a*]isoquinolin-2-yl)methanimine)

^{1}H NMR (600 MHz, $CDCl_3$)

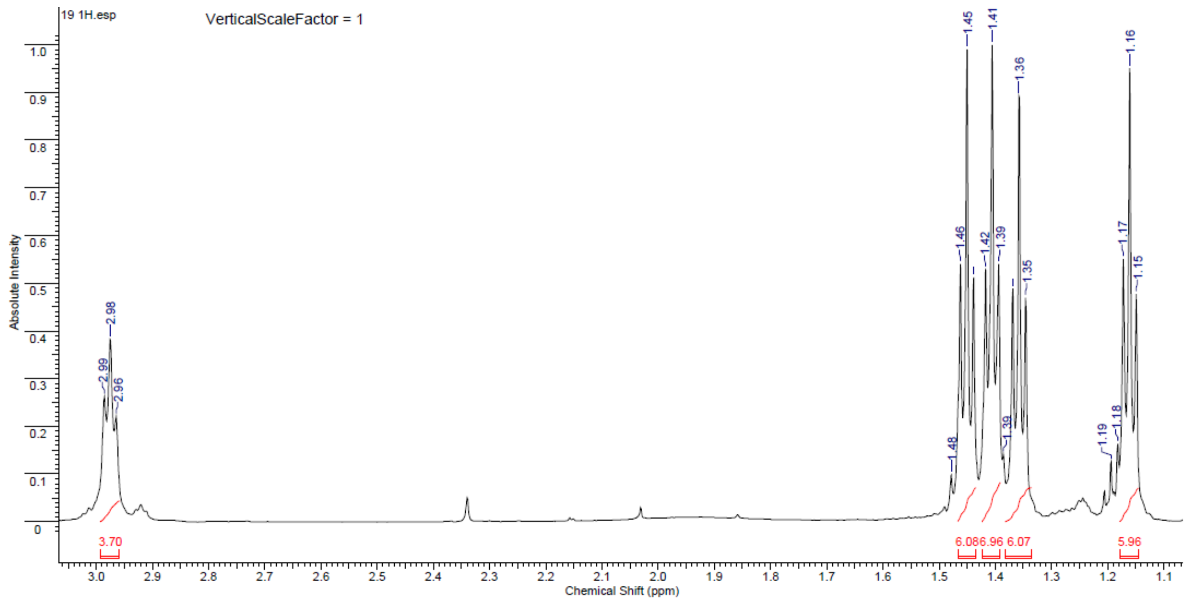

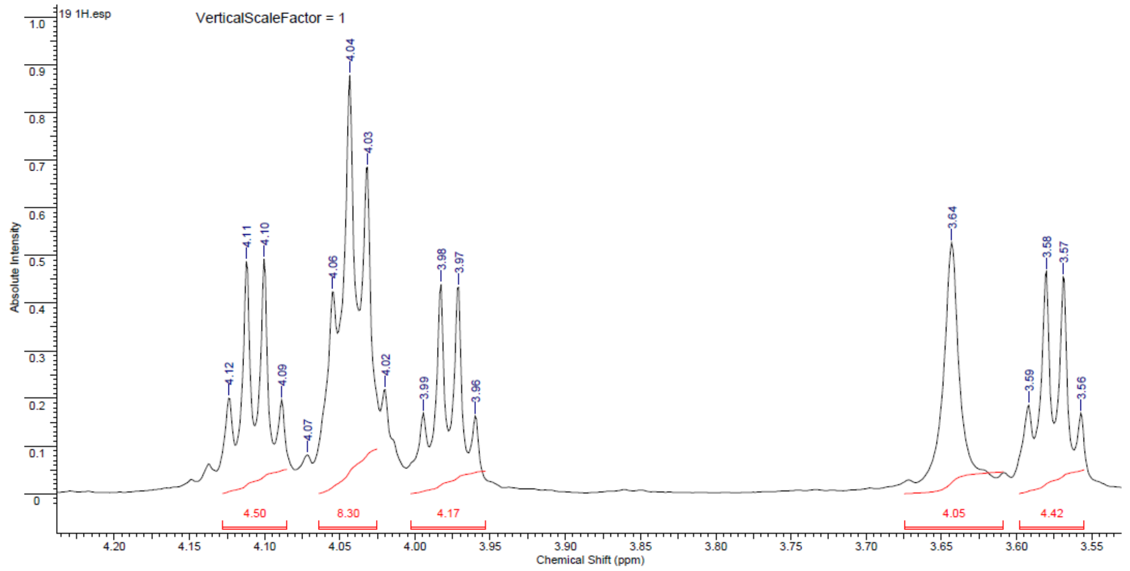

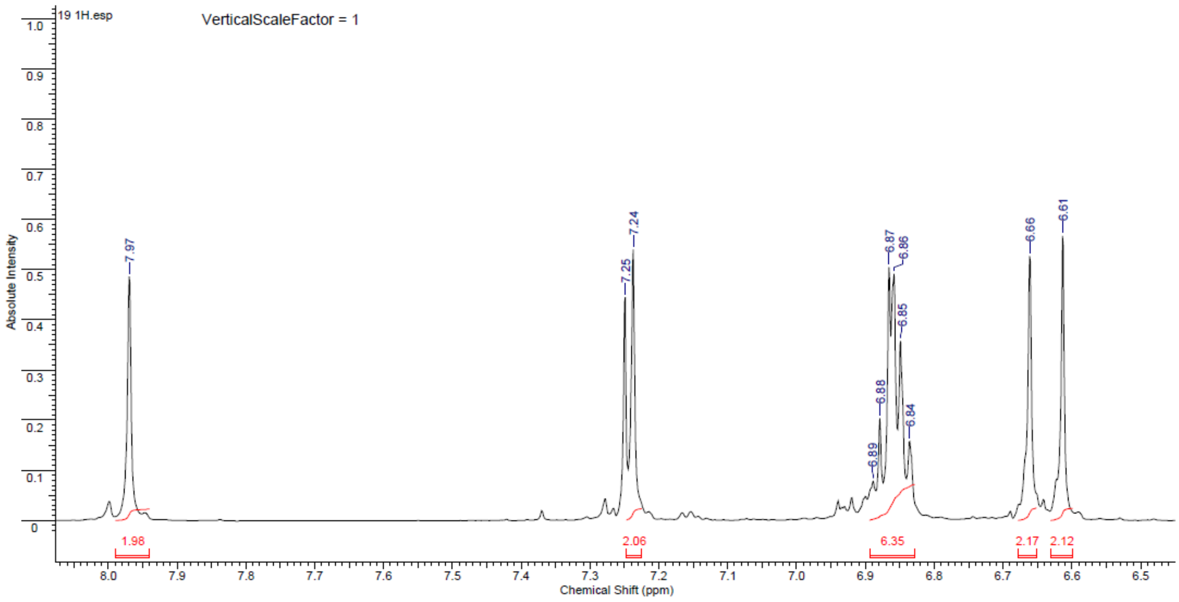

^{13}C NMR (150 MHz, DMSO-d_6)

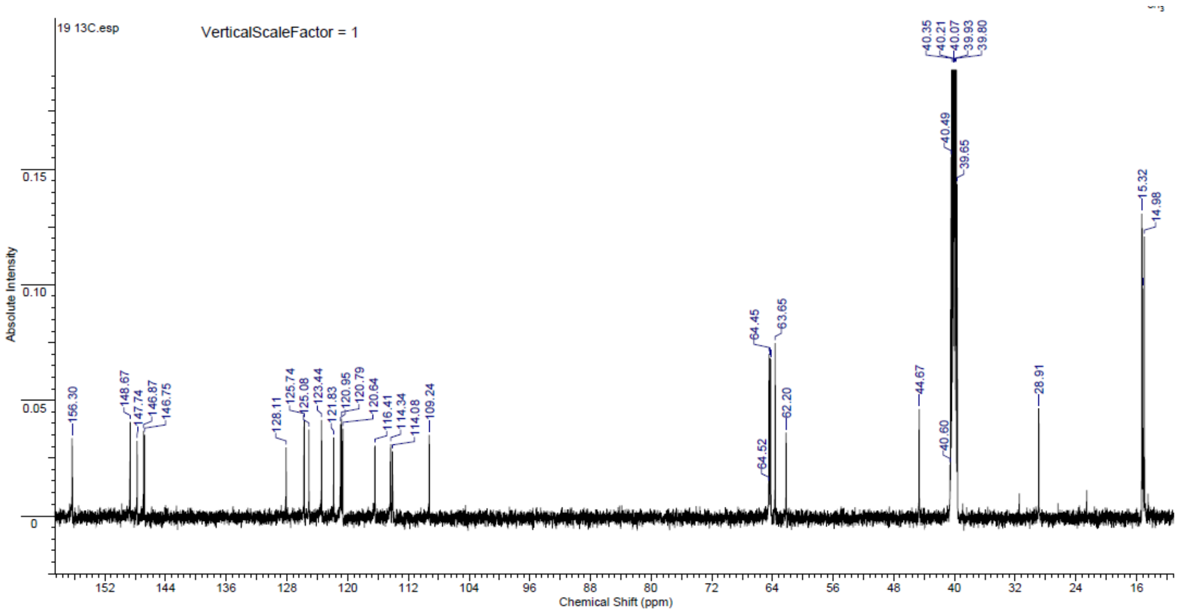

Multi-fingerprint Similarity Search aLgorithm (MuSSeL) outputs

Methods

The Multi-fingerprint Similarity Search aLgorithm (MuSSeL) is released as a ligand-based predictive web server to find putative protein drug targets of new conceived small molecules or to repurpose existing bioactive compounds [1, 2]. Predictions are computed by screening a collection including 611333 small molecules provided with high quality experimental bioactivity data covering 3357 protein drug targets, which were rationally selected from the latest release of ChEMBLdb (version 25, March 2019) [3]. Upon the request of a free licence, MuSSeL is publicly available at http://mussel.uniba.it:5000/.

MuSSeL has been interrogated to prioritize targets and biological activities of unsubstituted Schiff bases of 5,6-dihydro-1-phenylpyrrolo[2,1-*a*]isoquinoline (DHPPIQ) 2-carbaldehyde (Figure S1).

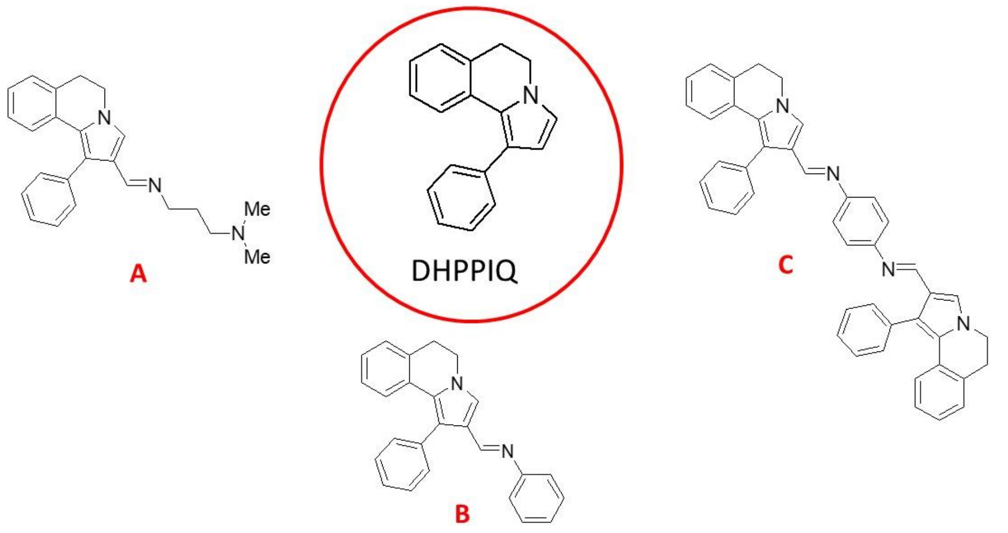

Figure S1. Core structure (DHPPIQ) and Schiff bases' scaffolds examined by MuSSeL.

[1] Montaruli, M.; Alberga, D.; Ciriaco, F.; Trisciuzzi, D.; Tondo, A.R.; Mangiatordi, G.F.; Nicolotti, O. Accelerating drug discovery by early protein drug target prediction based on multi- fingerprint similarity search. *Molecules* **2019**, *24*, 2233.

[2] Alberga, D.; Trisciuzzi, D.; Montaruli, M.; Leonetti, F.; Mangiatordi, G.F.; Nicolotti, O. A new approach for drug target and bioactivity prediction: The Multi-fingerprint Similarity Search aLgorithm (MuSSeL). *J. Chem. Inf. Model.* **2019**, *59*, 586-596.

[3] Gaulton, A.; Hersey, A.; Nowotka, M.; Bento, A.P.; Chambers, J.; Mendez, D.; Mutowo, P.; Atkinson, F.; Bellis, L.J.; Cibrián-Uhalte, E.; Davies, M.; Dedman, N.; Karlsson, A.; Magariños, M.P.; Overington, J.P.; Papadatos, G.; Smit, I.; Leach, A.R. The ChEMBL database in 2017. *Nucleic Acids Res.* **2017**, *45*(D1), D945-D954.

OUTPUTS

DHPPIQ

Prediction for DHPPIQ: c4ccc(c2ccn3CCc1ccccc1c23)cc4

Position	Target	Score	Reliability	Similars
1	Cyclooxygenase-2, Mus musculus	54.813 % (7.126)	YES	4
2	Cyclooxygenase-1, Mus musculus	54.813 % (7.126)	YES	4
3	Cyclin-dependent kinase 4/cyclin D1, Homo sapiens	39.660 % (5.156)	YES	8
4	Alpha-2a adrenergic receptor, Bos taurus	32.373 % (4.209)	YES	6
5	Alpha-1a adrenergic receptor, Bos taurus	32.373 % (4.209)	YES	8
6	Acetylcholinesterase, Electrophorus electricus	32.153 % (4.180)	NO	10
7	Cytochrome P450 19A1, Homo sapiens	30.919 % (4.019)	NO	11
8	Serotonin 6 (5-HT6) receptor, Homo sapiens	28.910 % (3.758)	NO	9
9	Cholinesterase, Equus caballus	28.873 % (3.754)	NO	11
10	Aryl hydrocarbon receptor, Homo sapiens	28.779 % (3.741)	NO	6
11	Serotonin 2a (5-HT2a) receptor, Homo sapiens	28.516 % (3.707)	NO	10
12	Serotonin 2c (5-HT2c) receptor, Homo sapiens	28.077 % (3.650)	NO	11
13	Sigma opioid receptor, Rattus norvegicus	27.605 % (3.589)	NO	11
14	Serotonin 2a (5-HT2a) receptor, Rattus norvegicus	27.433 % (3.566)	NO	11
15	Cannabinoid CB2 receptor, Homo sapiens	26.868 % (3.493)	NO	11
16	Serotonin 2b (5-HT2b) receptor, Homo sapiens	26.867 % (3.493)	NO	11

Compound A

Prediction for Compound A: CCOc4ccc(c2c(/C=N/CCCN(C)C)cn3CCc1cc(OCC)c(OCC)cc1c23)cc4OCC

Position	Target	Score	Reliability	Similars
1	Acetylcholinesterase, Electrophorus electricus	28.053 % (3.647)	NO	10
2	Butyrylcholinesterase, Equus caballus	27.638 % (3.593)	NO	9
3	Cholinesterase, Equus caballus	26.849 % (3.490)	NO	10
4	Multidrug resistance-associated protein 4, Homo sapiens	22.808 % (2.965)	NO	6
5	Bile salt export pump, Homo sapiens	22.808 % (2.965)	NO	7
6	Acetylcholinesterase, Homo sapiens	21.862 % (2.842)	NO	12
7	HERG, Homo sapiens	21.703 % (2.821)	NO	11
8	Butyrylcholinesterase, Homo sapiens	21.638 % (2.813)	NO	10
9	Neuronal acetylcholine receptor; alpha4/beta2, Homo sapiens	18.829 % (2.448)	NO	9
10	Dopamine D2 receptor, Homo sapiens	18.408 % (2.393)	NO	11
11	Dopamine D3 receptor, Homo sapiens	18.408 % (2.393)	NO	11
12	Serotonin 6 (5-HT6) receptor, Homo sapiens	18.142 % (2.358)	NO	11
13	Neuronal acetylcholine receptor; alpha4/beta2, Rattus norvegicus	17.996 % (2.339)	NO	11
14	Histamine H3 receptor, Homo sapiens	17.765 % (2.309)	NO	11
15	Phosphodiesterase 10A, Homo sapiens	17.738 % (2.306)	NO	8
16	Serotonin 1a (5-HT1a) receptor, Rattus norvegicus	17.734 % (2.305)	NO	12

Compound B

Prediction for Compound B: COc5ccc(/N=C/c3cn2CCc1ccccc1c2c3c4ccccc4)cc5

Position	Target	Score	Reliability	Similars
1	Acetylcholinesterase, Homo sapiens	28.200 % (3.666)	NO	5
2	Phosphodiesterase 10A, Homo sapiens	28.090 % (3.652)	NO	13
3	Potassium-transporting ATPase alpha chain 1, Sus scrofa	23.037 % (2.995)	NO	6
4	Tyrosine-protein kinase SRC, Homo sapiens	22.466 % (2.921)	NO	12
5	Acetylcholinesterase, Electrophorus electricus	22.057 % (2.867)	NO	11
6	Voltage-gated potassium channel subunit Kv1.5, Homo sapiens	21.654 % (2.815)	NO	9
7	Corticotropin releasing factor receptor 1, Homo sapiens	21.498 % (2.795)	NO	10
8	Serotonin 7 (5-HT7) receptor, Rattus norvegicus	21.453 % (2.789)	NO	7
9	Phosphodiesterase 3A, Homo sapiens	21.058 % (2.737)	NO	9
10	Cannabinoid CB2 receptor, Homo sapiens	21.057 % (2.737)	NO	10
11	Phosphodiesterase 7A, Homo sapiens	21.010 % (2.731)	NO	7
12	Histamine H3 receptor, Homo sapiens	18.070 % (2.349)	NO	12
13	Serotonin 6 (5-HT6) receptor, Homo sapiens	18.061 % (2.348)	NO	12
14	Cholinesterase, Equus caballus	17.962 % (2.335)	NO	10
15	Protein RecA, Mycobacterium tuberculosis	17.941 % (2.332)	NO	8
16	Serotonin transporter, Homo sapiens	17.897 % (2.327)	NO	11

Compound C

Prediction for Compound C: C(=N\c5ccc(/N=C/c3cn2CCc1ccccc1c2c3c4ccccc4)cc5)/c8cn7CCc6ccccc6c7c8c9ccccc9

Position	Target	Score	Reliability	Similars
1	Acetylcholinesterase, Electrophorus electricus	33.818 % (4.396)	YES	12
2	Cholinesterase, Equus caballus	29.622 % (3.851)	NO	11
3	Butyrylcholinesterase, Equus caballus	29.060 % (3.778)	NO	11
4	Telomerase reverse transcriptase, Homo sapiens	22.801 % (2.964)	NO	13
5	Serotonin 6 (5-HT6) receptor, Homo sapiens	22.754 % (2.958)	NO	13
6	Monoamine oxidase A, Homo sapiens	19.244 % (2.502)	NO	11
7	Monoamine oxidase B, Homo sapiens	19.244 % (2.502)	NO	12
8	HERG, Homo sapiens	18.348 % (2.385)	NO	11
9	Acetylcholinesterase, Bos taurus	18.158 % (2.360)	NO	11
10	Dopamine D4 receptor, Homo sapiens	17.777 % (2.311)	NO	10
11	Cyclin-dependent kinase 4/cyclin D1, Homo sapiens	17.024 % (2.213)	NO	11
12	Butyrylcholinesterase, Homo sapiens	16.393 % (2.131)	NO	13
13	Acetylcholinesterase, Rattus norvegicus	16.393 % (2.131)	NO	12
14	Acetylcholinesterase, Homo sapiens	16.316 % (2.121)	NO	10
15	Hepatocyte growth factor receptor, Homo sapiens	15.732 % (2.045)	NO	12
16	Sigma opioid receptor, Homo sapiens	14.256 % (1.853)	NO	12
